# Advances in Drug Delivery Integrated with Regenerative Medicine: Innovations, Challenges, and Future Frontiers

**DOI:** 10.3390/pharmaceutics17040456

**Published:** 2025-04-01

**Authors:** Manoochehr Rasekh, Muhammad Sohail Arshad, Zeeshan Ahmad

**Affiliations:** 1 College of Engineering, Design and Physical Sciences, Brunel University of London, Uxbridge UB8 3PH, UK; 2 Faculty of Pharmacy, Bahauddin Zakariya University, Multan 60800, Pakistan; 3 Leicester School of Pharmacy, De Montfort University, Leicester LE1 9BH, UK

**Keywords:** regenerative medicine, stem cells, drug delivery systems, controlled released, bioprinting

## Abstract

Advances in drug delivery systems adapted with regenerative medicine have transformed healthcare by introducing innovative strategies to treat (and repair in many instances) disease-impacted regions of the human body. This review provides a comprehensive analysis of the latest developments and challenges in integrating drug delivery technologies with regenerative medicine. Recent advances in drug delivery technologies, including the design of biomaterials, localized delivery techniques, and controlled release systems guided by mathematical models, are explored to illustrate their role in enhancing therapeutic precision and efficacy. Additionally, regenerative medicine approaches are analyzed, with a focus on extracellular matrix components, stem cell-based therapies, and emerging strategies for organ regeneration in both soft and hard tissue and in vitro model engineering. In particular, the review also discusses the applications of cellular components, including stem cells, immune cells, endothelial cells, and specialized cells such as chondrocytes and osteoblasts, and highlights advancements in cell delivery methods and cell–cell interaction modulation. In addition, future directions and pivotal trends emphasizing the importance of interdisciplinary collaboration and cutting-edge innovations are provided to address successful therapeutic outcomes in regenerative medicine.

## 1. Introduction

Advanced drug delivery technologies form the cornerstone of modern pharmaceutical research, offering superior efficacy compared to conventional drug formulations, especially in the domains of cancer therapy and tissue regeneration. Novel drug carriers bridge to meet two essential criteria: delivering medication precisely to the intended site within the body, aligning with its needs, and directly monitoring the drug’s activity during treatment. In contrast, the term “drug delivery system” refers specifically to a method of transporting drugs to a targeted area for defined durations. The primary goal behind advancing novel drug delivery systems is to ensure sustained and controlled drug release, maintaining optimal drug levels while minimizing side effects [1,2].

Amongst the various developments within this remit, ranging from materials to externally triggered devices, the size of (or a key component of some drug delivery systems) has led to monumental exploratory achievements, certainly on the ‘nano’ scale. Nanotechnology holds immense potential to transform the landscape of cancer detection and treatment, or perhaps even in sync, in the foreseeable future. Nanoparticles (NPs) have been shown to effectively penetrate biological barriers, target tumors, and selectively identify individual cancer cells for diagnosis and treatment [3,4]. As a result, nanomedicines not only enhance the therapeutic efficacy of drugs but also mitigate their adverse effects on healthy tissues. While significant progress has been made in the field of cancer nanomedicine, there is a growing interest in applying nanotechnology to other medical conditions, such as cardiovascular diseases, by enabling targeted delivery of anti-inflammatory agents, stabilizing vulnerable plaques, and promoting vascular repair. These advancements highlight the potential of nanotechnology in addressing complex pathologies beyond oncology [5]. As such, pharmaceutical companies are focusing on novel drug delivery systems to overcome bottlenecks and limitations of conventional drug delivery methods. The demand for high-performance, flexible, and controlled-release systems is being driven by advancements in patient compliance, clinical efficacy, prolonged product life, and economic benefits. Consequently, novel drug delivery systems are expected to be one of the fastest-growing segments within the healthcare sector [6].

An emerging component within drug delivery is the exploration of innovative technologies for regenerative medicine. Nanotechnology intended for the delivery of regenerative medicine has emerged as a transformative approach, offering precise control over the release of bioactive molecules and enhancing cell-material interactions to promote tissue repair. Current strategies in tissue engineering, which include the integration of nanoscale scaffolds, nanoparticles for drug and growth factor delivery, and nanofibers for structural support, have achieved notable clinical success and have been comprehensively reviewed by researchers [7]. It is well known that biomaterials play a crucial role in addressing a significant challenge in formulating and delivering protein and peptide biotherapeutics, namely their rapid degradation. Materials such as polyethylene glycol (PEG) for protein conjugation, poly (lactic-co-glycolic acid) (PLGA) for sustained-release nanoparticles, and hydrogels for localized delivery have demonstrated the ability to protect these biomolecules from enzymatic degradation while maintaining their bioactivity. These advancements ensure effective and controlled delivery of therapeutic proteins and peptides to target sites [8]. Drug delivery systems have been engineered to shield biomolecules from degradation in biological settings, enhancing their effectiveness while minimizing harmful side effects. For instance, encapsulating growth factors in biodegradable polymers like PLGA protects them from enzymatic degradation while ensuring sustained release. Similarly, combining drug delivery systems with stem cells offers a way to enhance delivery outcomes, focusing on improving transplanted cell survival, differentiation, and integration into host tissues. Recent advancements, such as hydrogel-based cell carriers and microparticle-laden scaffolds, show promise in circumventing such challenges by offering customizable properties to regulate cell behavior, promote vascularization, and modulate the immune response [9]. As technology progresses, more sophisticated drug delivery systems could pave the way for developing platforms to generate complex tissues, facilitating improved in vivo regeneration. Looking ahead, this progress would enable the creation of fully implantable organs tailored to the individual patient needs [4,10].

Regenerative medicine, a field of scientific inquiry, focuses on replacing damaged or lost tissues or organs caused by disease, injury, or birth defects [11]. It offers the potential to address healing challenges associated with various conditions once deemed incurable. In doing so, regenerative medicine addresses limitations associated with traditional transplantation therapy, including donor tissue shortage and the risk of immune rejection [12]. To achieve these goals, regenerative medicine employs diverse strategies such as cell therapy and tissue engineering, alongside the targeted delivery of therapeutic agents like drugs, proteins, and genes to the affected tissue site, aiding in the repair and healing processes [13]. Regenerative medicine harnesses the essential components of tissue regeneration, the extracellular matrix (ECM), cells, and various signaling molecules, either individually or in combination [14]. When regenerative potential is compromised by aging or systemic health issues, direct injection of regenerative factors into the affected site is often recommended. However, this approach often proves ineffective due to the rapid diffusion of therapeutic agents from the target site or their swift enzymatic deactivation, leading to suboptimal outcomes [15]. Recent advancements in drug discovery and biotechnology have introduced macromolecules, such as peptides, proteins, and nucleic acids, that exhibit poor solubility and short biological half-lives, requiring frequent administration to maintain therapeutic levels [16,17]. Hence, an optimal delivery system is imperative to safeguard therapeutic agents from degradation, enabling controlled and therapeutic delivery.

Drug delivery scaffolds are emerging as promising solutions, optimizing the therapeutic impact of drugs and bioactive substances while ensuring safety. These scaffolds not only enable precise delivery to target tissues or organs but also regulate the drug’s distribution and dosage within the body, thereby enhancing effectiveness and reducing potential side effects [18,19]. Effective delivery methods must account for the pharmacokinetics of the drug, encompassing its distribution, metabolism, and pharmacodynamics. Given the diverse physicochemical properties of active pharmaceutical ingredients (API), a thorough comprehension of materials science, formulation development, and manufacturing technologies is essential to ensure appropriate dosage forms are engineered. The focus on non-invasive administration routes, including oral, transdermal, inhalation, and mucosal delivery, has driven innovation in drug delivery strategies. These advancements have enhanced our understanding of drug kinetics and enabled the development of systems capable of overcoming biological barriers for more effective and patient-friendly therapies [20,21].

Regenerative medicine utilizes fundamental components such as the ECM, cells, and signaling molecules to support tissue regeneration [11]. Nevertheless, factors like aging or systemic health conditions may impair the body’s inherent regenerative capacities, prompting the need for interventions such as the localized administration of regenerative factors to affected areas [22,23]. This review delves into the pivotal role of advanced drug delivery systems in optimizing regenerative medicine approaches. By exploring innovative delivery technologies, we highlight how such integrations could foster tissue repair, wound healing, and the management of chronic diseases, offering precision and efficiency in therapeutic interventions.

Whilst broad, drug delivery systems that facilitate the regenerative medicine approach center on addressing specific challenges such as controlled release, bioactive scaffolds and patches, immune modulation, multi-agent delivery, sustained drug-cell therapies, and smart delivery systems. Controlled release ensures a steady and prolonged supply of therapeutic agents, while bioactive scaffolds and wound-healing patches provide structural support and enhance cellular interactions. Immune modulation directs the body’s immune response to create a conducive healing environment, and multi-agent delivery systems combine drugs, growth factors, and regenerative cells for synergistic effects. Advanced techniques, such as sustained drug-cell therapies, deliver both regenerative cells and therapeutic agents in tandem, while smart delivery systems, responsive to physiological cues, ensure precise targeting and on-demand release.

**Figure 1:**
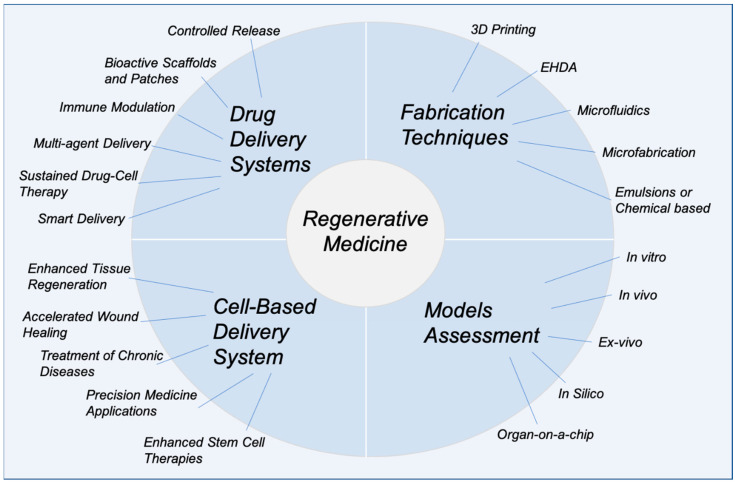
Overview of advanced drug delivery systems and cell-based delivery approaches integrated with regenerative medicine, highlighting key features, fabrication techniques, and model assessment methods (the figure was designed by the authors).

Cell-based delivery systems, in contrast, place an emphasis on the biological aspect of regenerative medicine, with applications such as enhanced tissue regeneration, accelerated wound healing, and precision medicine on an individual patient basis. These systems support the treatment of chronic diseases by improving the survival and functionality of therapeutic cells and by incorporating enhanced stem cell therapies for targeted tissue repair. Fabrication techniques like 3D printing, electrohydrodynamic atomization (EHDA), microfluidics, microfabrication, and emulsions or chemical-based approaches [4,7,8,17] are fundamental in creating such systems, offering unparalleled control over structure and composition. Model assessments, including in vitro, in vivo, ex vivo, in silico, and organ-on-a-chip methods, further enable a comprehensive evaluation of these delivery platforms, ensuring their safety, efficacy, and translational potential at multiple time points during therapy development (Figure 1). This review aims to provide an updated and comprehensive overview of recent advances in drug delivery systems and regenerative medicine, with a particular focus on emerging technologies such as bioprinting, stem cell therapies, and smart nanocarriers. Unlike previous reviews, this work integrates both technological innovations and clinical translation perspectives, while also addressing key challenges and limitations, such as biological barriers, safety concerns, and regulatory hurdles that impact the successful application of these therapies. The literature reviewed includes studies published primarily between 2014 and 2024, along with selected foundational works to provide broader context and deeper insights into the field.

## 2. Fundamentals of Regenerative Medicine

Regenerative medicine holds the promise of repairing or replacing damaged tissues and organs caused by aging, illness, or injury, as well as addressing congenital abnormalities. Encouraging preclinical and clinical findings indicate clear potential to address both chronic conditions and sudden injuries, spanning various organ systems and conditions such as skin wounds, heart illnesses (e.g., myocardial infarction repair), traumatic injuries, certain cancers, and many more. The realm of regenerative medicine encompasses a diverse array of approaches, including the utilization of materials and newly generated cells, often in combination, to substitute missing tissue, restoring both its structure and function, or aiding in tissue repair. While the body’s natural healing mechanisms can be harnessed to stimulate regeneration, adult humans have limited regenerative abilities compared to certain lower vertebrates [24]. Currently, organ and tissue transplantation, while effective, remains challenging due to donor shortage and immune-related complications. Regenerative medicine approaches offer potential solutions to overcome these obstacles [19,25].

### 2.1. Extracellular Matrix (ECM)

Essentially, the body consists of three components: cells, the extracellular matrix (ECM), which acts as a natural scaffold for cell proliferation and differentiation, and signaling molecules. The ECM, composed of proteins such as collagen, elastin, fibronectin, and laminin, along with glycosaminoglycans (GAGs) and proteoglycans, provides structural support and anchorage for cells. It regulates cell behavior, including polarity, differentiation, adhesion, and migration, while maintaining tissue and organ architecture. It is crucial for growth, regeneration, and inherent healing processes whilst maintaining structural integrity. Additionally, the ECM facilitates the exchange of metabolites, ions, and water. It is a complex network of biomolecules, including collagen, elastin, laminin, fibronectin, and GAGs, that biomechanically directs cell behavior, playing a crucial role in tissue function and homeostasis. Tissue regeneration can potentially be achieved using these elements either individually or in combination. However, successful tissue regeneration is not guaranteed by merely combining these elements; it requires a strategic biomedical approach to effectively integrate such biomaterials [26]. The ECM plays a crucial role in drug delivery systems by influencing drug penetration, retention, and efficacy. It serves as a structural network of proteins and polysaccharides, regulating cellular interactions and acting as a physical barrier to drug diffusion within tissues, particularly in tumors. Modulating the ECM, such as reducing its density or altering its composition, can improve drug delivery by enhancing permeability and reducing interstitial pressure, particularly by targeting components like collagen and hyaluronic acid, which contribute to ECM stiffness and resistance. Furthermore, specific ECM components, such as integrin, fibronectin, or proteoglycans, can be targeted to enhance the specificity of drug delivery systems, including nanoparticle-based therapies, enabling efficient and localized treatment.

The ECM not only offers physical support for cells, but it also creates a natural environment for cell proliferation and differentiation, commonly known as morphogenesis, which aids in tissue regeneration and organogenesis [27]. Regenerating and repairing major tissue defects with cell supply alone is challenging due to the loss of both cells and the ECM. To promote tissue regeneration at a defective biological site, a three-dimensional (3D) scaffold of artificial ECM can be used to create a ‘similar’ environment for cells to attach, grow, and differentiate. If the artificial ECM is biologically compatible, cells around the scaffold will infiltrate and multiply, leading to differentiation.

Biomaterials such as collagen, gelatin, chitosan, and PLGA play a crucial role in creating cell scaffolds. These scaffolds must be porous and biodegradable. The porous structure allows cells to infiltrate and access essential oxygen and nutrients, supporting cell-based tissue regeneration and facilitating the formation of a natural ECM. However, if the scaffold remains for too long, it may physically hinder tissue regeneration. Therefore, it is essential to control both the timing and structure of scaffold biodegradation at the defect site for successful tissue regeneration [27,28].

If the tissue surrounding the defect lacks the ability to regenerate, relying only on the scaffold may not always result in successful regeneration. The scaffold should be utilized in combination with cells, such as stem cells or fibroblasts, which proliferate and differentiate to rebuild tissue, and signaling molecules, including growth factors such as bone morphogenetic proteins (BMPs) and Vascular Endothelial Growth Factor (VEGF) that stimulate cell growth and angiogenesis, cytokines that regulate immune responses and inflammation, and chemokines that guide cell movement to the injury site for effective repair. Cells with high proliferation and differentiation potential are administered to tissue defects to promote regeneration. Direct infusion of a growth factor into a regenerating site is typically ineffective, as it may degrade rapidly or diffuse away before exerting its effects. However, in certain instances, such as acute injuries requiring immediate stimulation, poorly vascularized tissues where scaffold-based delivery is insufficient, or critical-size defects needing high local concentrations, direct infusion may be necessary. This inefficacy arises because the growth factor quickly diffuses from the injection site and is either enzymatically digested or deactivated. For the growth factor to function effectively, a technology is needed to ensures its stability, protects it from degradation, and provides controlled, localized release at the target site. This represents the second key innovation in tissue engineering and regenerative medicine: advanced drug delivery systems [26].

**Figure 2:**
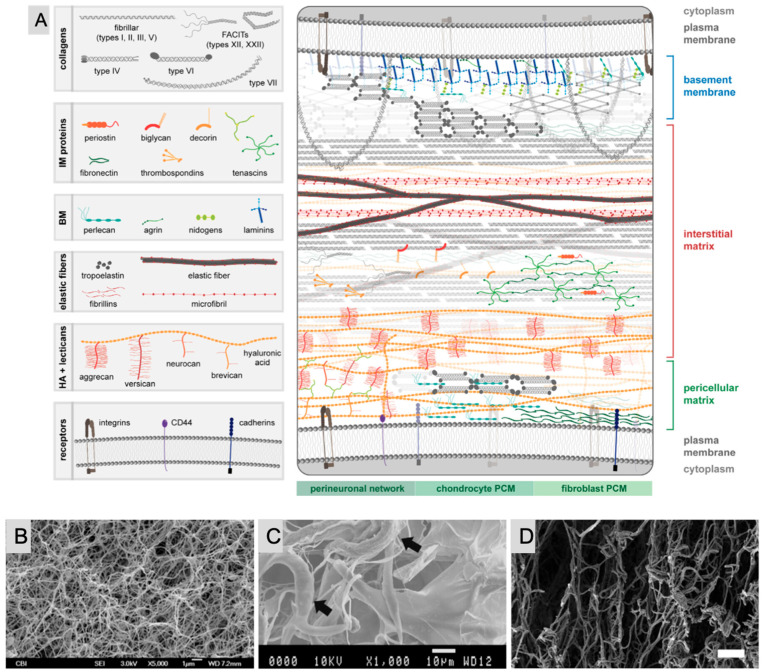
(**A**) Distribution of key ECM components at the cellular level. The top cell is an epidermal or endothelial cell separated from the interstitial matrix (IM) by the basement membrane (BM). The arrangement of ECM is constant across the native BM, but in the schematic, components are minimized or removed to highlight the relative distribution of BM protein. The BM is connected to the IM through type VII collagen anchoring fibrils and/or type VI collagen. The pericellular matrix (PCM) surrounding the bottom cell is arranged to show differences between the PCM of neurons (perineuronal nets), chondrocytes, and fibroblasts. Reprinted from reference [26] with permission from Elsevier (not to scale). SEM images of biomaterials composed of (**B**) a purified single ECM protein (pure collagen I hydrogel). Reprinted from reference [30] with permission from Elsevier. (**C**) Multiple ECM proteins (crosslinked collagen I-elastin scaffold). Arrows indicate points of interaction between collagen and elastin fibers. Reprinted from reference [31] with permission from Elsevier. (**D**) Tissue-derived materials (decellularized human adipose ECM hydrogel). Reprinted with permission from reference [32]. Copyright (2014) John Wiley & Sons, Inc.

Despite substantial research, the ECM is frequently oversimplified as a uniform network, but in reality, it is made up of heterogeneous fibrillar networks inside an amorphous matrix that varies significantly among tissues. The ECM is made up of GAGs and proteins such as glycoproteins and proteoglycans. Hyaluronic acid is a typical GAG, but others are smaller, sulfated, and linked to proteins, forming proteoglycans that play diverse roles in cell signaling and ECM construction. Glycoproteins such as fibronectin and laminin are essential for ECM formation and cell interactions. Additionally, the ECM regulates cell and tissue homeostasis through biomechanical signaling. Injury, genetic abnormalities, or disease can cause ECM dysregulation, profoundly impacting cell behavior. Understanding pathological alterations in ECM composition is critical for developing in vitro disease models (Figure 2A) [26,29]. Biomaterials shown in Figure 2B–D include a purified single ECM protein (pure collagen I hydrogel), multiple ECM proteins (crosslinked collagen I-elastin scaffold), and tissue-derived materials (decellularized human adipose ECM hydrogel) (Figure 2B–D) [30,31,32]. These examples emphasize the importance of biomaterial innovations in mimicking the complexity of the ECM to advance tissue engineering and regenerative medicine.

### 2.2. Cellular Components: Cells Used in Regenerative Medicine

#### 2.2.1. Stem Cells

##### Embryonic Stem Cells (ESCs)

Embryonic stem cells have been widely explored for their potential applications in regenerative medicine. These are pluripotent stem cells that have the potential for infinite growth. They are produced from the embryonic (blastocyst stage) inner cell mass and express several distinct cell surface markers. Because of their pluripotency, ESCs have been used in a wide range of clinical and preclinical research involving spinal injury, cardiovascular disease, and other neurodegenerative illnesses [33,34]. Although there is significant interest in the potential applications of ESCs in both veterinary and human medicine, their use is entangled in political and ethical debates due to the necessity of deriving these cells from live human embryos [34,35]. These controversies, along with standard scientific inquiry, have led to an extensive investigation into adult stem cells, focusing on their abilities for self-renewal and differentiation into various cell types. These studies have revealed that hormetic dose responses are consistently observed across all extensively researched adult stem cells, displaying remarkably uniform characteristics regardless of the stem cell types and inducing substances examined [36,37].

##### Adult Stem Cells

Adult stem cells are undifferentiated cells present in all organs of the body. Typically maintained in an inactive, non-dividing state, these cells can divide and differentiate to replace naturally dying cells within their tissue and repair wounds in response to injury. Due to their proliferation and tissue-regenerating abilities, adult stem cells hold promise for treating a wide range of degenerative disorders and conditions associated with aging. For example, mesenchymal stem cells (MSCs) have been used in clinical studies to treat osteoarthritis, cardiovascular diseases, neurodegenerative disorders such as Parkinson’s disease, and age-related macular degeneration, demonstrating their therapeutic potential. Furthermore, since stem cells are often considered potential origins of malignant tumors, understanding the mechanisms that regulate their proliferative capacity could lead to new cancer therapies by identifying targets to control abnormal growth and prevent tumor formation [38].

Adult stem cells have shown great potential in emerging drug delivery systems due to their natural ability to home to sites of injury, inflammation, or tumors. These cells can be engineered to act as carriers for therapeutic agents, such as drugs, genes, or nanoparticles, ensuring targeted delivery with minimized off-target effects. Additionally, their immunomodulatory properties enhance biocompatibility, while their capacity for self-renewal supports sustained therapeutic outcomes. Adult stem cell-based systems are particularly valuable in regenerative medicine and targeted cancer therapies. Dysregulation of the mechanisms that keep stem cells in an inactive, non-proliferative state can result in cancer. This dysregulation may arise from mutations or alterations in key signaling pathways such as Wnt (Wingless/Int-1), Notch, and Hedgehog (Hh), which are critical for maintaining stem cell quiescence and self-renewal. Additionally, epigenetic modifications, such as aberrant DNA methylation or histone acetylation, and changes in the tumor microenvironment, including inflammatory cytokines and hypoxia, can further disrupt the delicate balance of stem cell regulation. This raises safety concerns about stem cell therapies but also presents opportunities for new cancer treatments. Gaining insight into molecular pathways that malfunction during a stem cell’s progression towards tissue development but lead to cancerous outcomes instead may be valuable, as new treatments could be developed. Although numerous clinical trials are investigating the use of adult stem cells to treat various diseases, only a few have led to approved therapies. One notable success is bone marrow transplantation, which uses hematopoietic stem cells (HSCs) to regenerate blood cells and is widely used to treat hematologic cancers and other disorders. Additionally, skin stem cell therapy has demonstrated significant efficacy in restoring damaged skin in burn victims, showcasing the therapeutic potential of stem cell-based approaches [38,39].

##### Induced Pluripotent Stem Cells (iPSCs)

Induced pluripotent stem cells are cells that originate from adult somatic cells that are genetically reprogrammed to an embryonic stem cell-like state by forcing the expression of genes and factors required to maintain ES cell-defining properties. Induced pluripotent stem cells (iPSCs) hold significant potential in drug delivery systems due to their ability to differentiate into various cell types and their capacity for patient-specific applications. Strategies to develop iPSCs typically involve reprogramming somatic cells using transcription factors such as Oct4 (Octamer-binding transcription factor 4), Sox2 [SRY (Sex-determining Region Y)-Box 2], Klf4 (Kruppel-like factor 4), and c-Myc (Cellular Myc), delivered through viral or non-viral vectors. IPSCs can be engineered to deliver therapeutic agents, such as anticancer drugs, proteins, or nanoparticles, directly to target tissues with high precision, as demonstrated in studies where iPSCs were used to deliver nanoparticles for cancer therapy. Their versatility enables personalized drug screening and regenerative therapies, while their self-renewal capability supports sustained delivery over time. Furthermore, iPSCs are invaluable for modeling diseases, such as neurodegenerative disorders or cardiovascular conditions, to evaluate and optimize drug delivery systems, thereby accelerating the development of more effective and targeted treatments.

Over a decade ago, researchers discovered that mature human somatic cells, such as skin fibroblasts or peripheral blood cells, could be reprogrammed into a pluripotent state using transcription factors (Oct4, Sox2, Klf4, and c-Myc), enabling their differentiation into various cell lineages. For instance, Takahashi and Yamanaka’s groundbreaking 2006 study [40] demonstrated the generation of iPSCs from mouse fibroblasts, followed by similar success with human cells in 2007 [41]. This breakthrough paved the way for personalized, cell-based autologous therapies for diseases such as Parkinson’s disease, where dopaminergic neurons derived from iPSCs have shown promise in preclinical studies. Furthermore, advancements in precise DNA editing technologies, such as CRISPR-Cas9 (clustered regularly interspaced short palindromic repeats-associated protein 9), have significantly amplified the potential of iPSC-based approaches by enabling patient-specific gene corrections. This has sparked growing interest in therapies for genetic disorders and degenerative diseases. However, to create optimized disease models for discovering new treatments, human patient-derived iPSCs must differentiate into cell states that accurately replicate the characteristics of diseased cells and tissues. Consequently, the clinical value of iPSC-derived products heavily relies on advancements in directed differentiation, cell state conversion, and tissue engineering [42,43].

**Figure 3:**
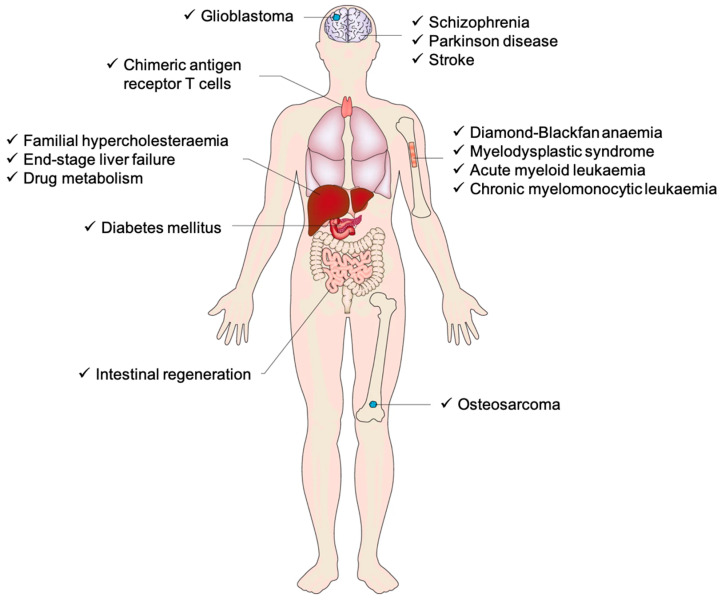
An overview of the current state of human induced pluripotent stem cell (iPSC) chimera models, demonstrating the range of tissues and organs modeled as chimeras, along with the disease classifications that have been modeled in chimeras [43].

Moreover, the creation of iPSCs involves reprogramming mature somatic cells, such as skin or blood cells, into a pluripotent state by introducing specific transcription factors mentioned above (Oct4, Sox2, Klf4, and c-Myc) through methods like viral vectors or RNA delivery. These factors reset the cells’ epigenetic state, enabling them to “de-differentiate” and regain the ability to develop into various cell types. The reprogrammed cells are then cultured to form iPSC colonies, which can be expanded and further differentiated into specific cell lineages for research or therapeutic purposes. Additionally, tissues derived from iPSCs closely match the cell donor, which is crucial for disease modeling and drug screening studies. It is expected that researchers will utilize iPSCs to learn how to reprogram cells to repair damaged tissues in the human body [42]. With the recent surge in cell-based therapies being investigated in preclinical and clinical settings for numerous diseases, iPSC-based chimeric disease models via xenotransplantation have become an effective way to drive these advancements by accurately replicating human diseases (Figure 3).

#### 2.2.2. Progenitor Cells

Progenitor cells are a type of biological cell that is more specialized than stem cells but still has the ability to differentiate into specific types of cell lines. Unlike stem cells, which are pluripotent and can give rise to many different cell types, progenitor cells are often committed to differentiating into a narrower subset of cells. These cells have a limited capacity for self-renewal compared to stem cells and are typically more involved in tissue repair and regeneration.

Progenitor cells play a crucial role in replenishing damaged tissues and supporting the healing process. They are found at various anatomical sites, including the bone marrow, skin, and liver. For instance, myeloid progenitor cells in the bone marrow give rise to red blood cells, platelets, and specific types of white blood cells, while neural progenitor cells contribute to the formation of neurons and glial cells in the brain [44,45]. With their limited differentiation potential, progenitor cells are essential for tissue maintenance and regeneration, particularly following injury. They can be activated by growth factors like fibroblast growth factors (FGFs) or Wnt proteins to promote their proliferation and differentiation. Transferring progenitor cells from one individual to another typically involves isolating them from sources such as bone marrow or cord blood, purifying them using techniques like flow cytometry, and preserving them for transplantation. For in vitro cultivation, progenitor cells are grown in specialized media enriched with cytokines and growth factors, such as interleukin-3 (IL-3) or stem cell factor, to support their proliferation and direct their differentiation into target cell types. Progenitor cells hold great promise in regenerative medicine, with ongoing research exploring their potential in treating conditions such as neurodegenerative disorders, heart disease, and diabetes, where targeted tissue regeneration is crucial.

#### 2.2.3. Somatic Cells

Somatic cells are non-reproductive cells that form the body’s tissues and organs, excluding sperm and egg cells, which are classified as germ cells. These cells are the foundation of most bodily functions and are essential for the body’s structure and operation. Somatic cells are diploid cells containing two sets of chromosomes, one inherited from each parent. They constitute the vast majority of an organism’s cells and are responsible for forming all tissues and organs. Examples of somatic cells include muscle cells, nerve cells, skin cells, and blood cells, which collectively perform the diverse functions necessary for maintaining the body’s structure and physiology. Unlike germ cells, which are responsible for reproduction and pass genetic information to offspring, somatic cells do not contribute to heredity. Instead, they undergo mitosis (a process of cell division) to replace old, damaged, or dead cells, helping maintain the health of tissues [46].

The human body contains hundreds of specialized somatic cells, each performing specific functions vital to overall health and operation. Epithelial cells line organ surfaces, providing protection while aiding in absorption and secretion. Muscle cells including skeletal, cardiac, and smooth types facilitate movement and support essential functions such as heart contractions and digestion. Neurons, found in the brain and throughout the nervous system, transmit electrical signals that regulate body functions and responses to external stimuli. Blood cells, consisting of red and white blood cells, circulate in the bloodstream; red blood cells transport oxygen, while white blood cells play a crucial role in the immune system by fighting infections. Together, these somatic cells ensure that the body functions efficiently.

Somatic cells carry out a variety of essential functions within the body, depending on their specific type and specialization. One of their primary roles is building and maintaining tissues, such as muscle, skin, and bone, which ensures the structural integrity of the body. Additionally, somatic cells are critical in supporting bodily functions. For example, neurons transmit electrical signals in the nervous system, while muscle cells facilitate movement. Somatic cells are also involved in healing and regeneration, playing a vital role in tissue repair. Skin cells, for instance, regenerate quickly to heal wounds, and liver cells possess the ability to regenerate after damage, contributing to the body’s recovery and overall maintenance. However, the lifespan and replacement rate of somatic cells vary significantly depending on their type and function. Some cells, such as skin cells, are replaced frequently due to their exposure to external factors and constant shedding. In contrast, other cells, like neurons, have a much longer lifespan and are rarely replaced after damage, often making injury to these cells more permanent. The ability of somatic cells to undergo mitosis, a process of cell division, enables tissues to continually renew and repair themselves. However, this regenerative capacity tends to diminish as the body ages, leading to slower healing and reduced cellular turnover in older individuals.

Somatic cells are gaining attention in drug delivery systems due to their potential to be modified as carriers for therapeutic agents. For example, red blood cells have been engineered to deliver anticancer drugs like doxorubicin, taking advantage of their biocompatibility and long circulation time. Similarly, mesenchymal stromal cells (MSCs) are being explored for their ability to home to inflamed or damaged tissues, where they can deliver therapeutic proteins or RNA molecules. These advancements highlight the versatility of somatic cells in targeted drug delivery, reducing off-target effects and improving treatment efficacy. Unlike stem cells, somatic cells are terminally differentiated, offering stability in their function and limiting undesired proliferation. They are particularly valuable in autologous therapies, where patient-derived somatic cells reduce the risk of immune rejection, making them a promising tool for personalized and targeted drug delivery strategies. Moreover, somatic cells play a pivotal role in medical treatments. In the case of cancer, mutations in somatic cells can lead to uncontrolled cell growth, resulting in the formation of tumors. Understanding the behavior of somatic cells is essential in developing cancer treatments like chemotherapy, which targets rapidly dividing cells. Additionally, somatic cells are fundamental in regenerative medicine, where they are manipulated to repair or replace damaged tissues or organs. Therapies such as skin grafts and stem cell treatments, which are often derived from somatic cells, highlight their potential in healing and tissue regeneration. In brief, somatic cells are the essential building blocks of the body’s tissues and organs. They are crucial for maintaining bodily functions, supporting growth, and enabling tissue repair. Their specialized nature ensures the proper functioning of each part of the body, making an understanding of somatic cells vital for advancements in medical science, particularly in fields like cancer research, regenerative medicine, and tissue engineering [46,47].

#### 2.2.4. Immune Cells

Immune cells play a vital role in defending the body against pathogens and maintaining overall immune health. Being part of the immune system means they are involved in recognizing, attacking, and eliminating foreign invaders like bacteria, viruses, and even cancerous cells. Among the key players in the immune response are T cells and dendritic cells, both of which have gained significant attention in recent years for their roles in immunotherapy and cancer treatment.

T cells, a type of white blood cell or lymphocyte, are essential to the adaptive immune response. They come in several forms, with cytotoxic T cells being particularly important for directly attacking infected or cancerous cells. Helper T cells assist other immune cells by releasing cytokines, which help regulate the immune response. Moreover, T cells are employed in immunotherapies like CAR-T cell therapy, where a patient’s T cells are genetically modified to target cancer cells specifically. This involves extracting the patient’s T cells, engineering them to express chimeric antigen receptors (CARs) that recognize specific proteins on the surface of cancer cells (e.g., CD19 in B-cell leukemia), and then reintroducing them into the patient. Once infused, these modified T cells can identify and destroy cancer cells with high precision, leading to remarkable success in treating cancers such as leukemia and lymphoma, with some patients achieving long-term remission [48].

Dendritic cells act as antigen-presenting cells, which means they process foreign substances and present them to T cells to initiate an immune response. These cells are essential for activating naive T cells and play a central role in linking the innate and adaptive immune systems. In the context of cancer immunotherapy, dendritic cell vaccines are being developed to enhance the body’s ability to fight tumors by priming T cells against cancer-specific antigens. Additionally, these cells are also being explored in regenerative immunology, where they help modulate immune responses in tissue regeneration and healing processes. Together, T cells and dendritic cells form a critical partnership in targeting diseased cells, particularly in cancer treatment. Their applications in immunotherapy represent a major advance in personalized medicine, offering new hope for patients with difficult-to-treat conditions [49].

Immune cells, particularly T cells and dendritic cells, play a critical role in drug delivery systems, especially in immunotherapy development. Targeting these cells allows drug delivery systems to enhance the body’s natural immune response against diseases such as cancer. For example, nanoparticles can be engineered to deliver drugs directly to T cells, improving their ability to recognize and destroy cancer cells. Similarly, dendritic cells can be targeted to enhance antigens presentation to T cells, increasing the effectiveness of vaccine-based therapies. These approaches are essential for developing more precise and effective treatments in immunotherapy [50].

#### 2.2.5. Endothelial Cells

Endothelial cells are specialized cells that form the inner lining of blood vessels, including arteries, veins, and capillaries. They create a thin, continuous layer called the endothelium, which plays a critical role in maintaining vascular health and regulating the passage of materials and nutrients between the bloodstream and surrounding tissues. These cells are essential for the function of the cardiovascular system, contributing to processes such as blood flow regulation, inflammation response, and blood clotting. Endothelial cells play a key role in angiogenesis, the formation of new blood vessels, which is essential for wound healing, organ growth, and tissue regeneration. In tissue engineering and regenerative medicine, these cells are crucial for ensuring engineered tissues receive sufficient blood supply to support cell survival and growth. Endothelial cells help form vascular networks that integrate with the body’s circulatory system, making them vital for the success of implantable tissue constructs [51]. For example, in the development of tissue-engineered organs or biomaterials, endothelial cells are seeded onto scaffolds to encourage the growth of blood vessels [51]. This process allows engineered tissue to vascularize, ensuring long-term viability and function post-implantation. Recent efforts are directed at bioactive coatings and growth factor-based therapies to enhance endothelial cell activity and improve angiogenesis in tissue-engineered constructs.

Endothelial cells are vital in tissue engineering, forming blood vessels, regulating barrier function, controlling molecular exchange, and responding to biochemical signals. These roles ensure nutrient delivery while blocking harmful substances, making them essential for functional implantable tissues [52].

Moreover, endothelial cells, which form the inner lining of blood vessels, are being extensively studied for their potential in targeted drug delivery systems. Their natural interaction with circulating substances makes them an ideal target for therapies aimed at treating vascular diseases, cancer, and inflammatory conditions. For instance, nanoparticle-based delivery systems, such as gold nanoparticles or polymeric nanoparticles, have been engineered to bind specifically to receptors like vascular cell adhesion molecule-1 (VCAM-1) on endothelial cells, enabling localized drug delivery to sites of vascular injury or tumor angiogenesis. Similarly, biodegradable polymers like PLGA and liposomes are often coated with targeting ligands such as antibodies or peptides to enhance binding and drug delivery to diseased tissues. These advanced systems not only improve the precision of drug delivery but also exploit endothelial cells’ role in regulating vascular permeability, ensuring therapeutic agents reach compromised regions with minimal off-target effects [53]. For example, endothelial cells in tumors exhibit abnormal behavior during tumor angiogenesis, providing a unique opportunity to target cancer cells via the vasculature. Drugs or nanoparticles can be designed to exploit this abnormal endothelial function, allowing for more efficient drug delivery into the tumor. This strategy is also being explored in cardiovascular diseases, where endothelial dysfunction plays a role in the development of atherosclerosis, enabling drug-loaded nanoparticles to treat inflamed or damaged blood vessels effectively [53,54,55].

#### 2.2.6. Chondrocytes and Osteoblasts

Chondrocytes and osteoblasts are two pivotal cellular components in regenerative medicine, particularly in the repair and regeneration of musculoskeletal tissues. Chondrocytes are specialized cells found in cartilage, responsible for maintaining the extracellular matrix by producing collagen and proteoglycans. These cells are central to the regeneration of articular cartilage, which has limited self-healing ability due to its avascular nature. In regenerative medicine, autologous chondrocyte implantation has emerged as a prominent therapeutic approach for treating cartilage defects, where chondrocytes are harvested, expanded, and re-implanted into damaged tissue to promote repair [56]. Osteoblasts, on the other hand, are key cells in bone formation. Derived from mesenchymal stem cells, osteoblasts synthesize the bone matrix and regulate mineralization, making them essential for the repair of bone defects and fractures. In clinical applications, osteoblasts are often used in combination with scaffolding materials and growth factors to enhance bone regeneration [57].

Integrating cellular therapies with advanced drug delivery systems holds great promise for enhancing regenerative outcomes. Controlled release systems can benefit chondrocytes and osteoblasts by delivering growth factors, anti-inflammatory agents, or therapeutic drugs directly to tissue repair sites. Drug delivery platforms such as hydrogels, nanoparticles, and scaffolds not only support cell proliferation but also provide sustained release of bioactive molecules that promote tissue regeneration. For example, BMPloaded scaffolds have successfully enhanced osteoblast differentiation and accelerating bone healing [56,57]. Combining cellular components with advanced drug delivery systems optimizes regenerative therapies, improving outcomes for patients with cartilage and bone injuries.

#### 2.2.7. Myocytes in Regenerative Medicine

Myocytes, also known as muscle cells, play a vital role in regenerative medicine, particularly in treating degenerative muscle diseases and injuries. These specialized cells are responsible for muscle contraction and are characterized by their elongated structure and ability to differentiate from satellite cells, which are essential for muscle repair. In regenerative medicine, myocytes are often used to restore damaged muscle tissue through cell-based therapies, such as myoblast transplantation. This approach involves injecting cultured myoblasts into the injured area to promote muscle regeneration and has been explored in conditions like muscular dystrophy and traumatic muscle loss [58]. Recent advancements in stem cell research have also highlighted the potential of iPSCs to differentiate into myocytes, offering new avenues for creating patient-specific regenerative therapies [59].

Integrating drug delivery systems with myocyte-based therapies holds significant potential for enhancing treatment efficacy. Targeted drug delivery platforms, such as hydrogels and nanoparticles, can be engineered to release growth factors, anti-inflammatory agents, or other bioactive molecules that support myocyte survival, proliferation, and differentiation. For instance, the sustained release of insulin-like growth factor (IGF-1) from biomaterials has been shown to promote myocyte regeneration and improve muscle repair [58]. Moreover, controlled-release systems provide localized and sustained drug delivery, minimizing systemic side effects and optimizing therapeutic outcomes in muscle regeneration. The combination of myocyte-based cell therapies with advanced drug delivery technologies represents a promising strategy for muscle tissue engineering and regenerative medicine. Table 1 summarizes key cellular components in regenerative medicine, their features, applications, and integration with advanced drug delivery systems.

### 2.3. Cell Sources and Applications

#### 2.3.1. Cell Sources

Sourcing cells for regenerative medicine is a well-established area of research and progression, as different types of cells exhibit unique properties that influence their therapeutic potential. Broadly, cell sources are categorized into autologous, allogeneic, and xenogeneic categories. Autologous cells are derived from the patient’s own body, minimizing the risk of immune rejection, and are commonly used in tissue repair therapies, such as skin grafts or cartilage regeneration. Allogeneic cells, harvested from donors, are more accessible but may present challenges such as immune rejection and the need for immunosuppressive therapies. Xenogeneic cells, derived from other species, like porcine cells, are explored primarily in experimental models but raise ethical and immunological concerns [60]. Stem cells, including MSCs from bone marrow, adipose tissue, and iPSCs, are among the most promising cell sources in regenerative medicine due to their multi-potency and ability to differentiate into various tissue types [61].

The integration of cell sources with advanced drug delivery systems is an emerging area of research. Controlled drug release technologies can be paired with cell-based therapies to enhance cell viability, promote differentiation, and improve therapeutic outcomes. For example, hydrogels and 3D-printed scaffolds loaded with growth factors or drugs can be used to deliver stem cells directly to the target site, providing a supportive environment that enhances cell survival and integration into the host tissue. In this way, the combination of cell sources with drug delivery platforms offers a promising avenue for addressing challenges like poor cell engraftment and enhancing tissue regeneration [61]. This synergy between cell-based therapies and drug delivery systems is essential for advancing regenerative medicine, as it not only ensures the sustained release of therapeutic agents but also creates a more conducive microenvironment for the transplanted cells. By delivering growth factors or cytokines in a controlled manner, these systems can stimulate cellular activities such as migration, proliferation, and differentiation at the target site. Furthermore, drug delivery platforms like nanoparticles or hydrogels can shield cells from hostile conditions, such as inflammation or oxidative stress, improving their integration into the host tissue and promoting long-term tissue regeneration.

#### 2.3.2. Cell Isolation and Culture

Cell isolation and culture are foundational techniques in regenerative medicine, allowing researchers to obtain, expand, and manipulate cells for therapeutic applications. Isolation methods vary depending on the cell source, with common techniques including enzymatic digestion, density gradient centrifugation, and magnetic-activated cell sorting. These methods ensure the extraction of viable and functionally relevant cells from tissues such as bone marrow, adipose tissue, or peripheral blood. Once isolated, cells are cultured under controlled conditions, optimizing factors like temperature, pH, and nutrient supply to promote cell growth and maintain their functional properties. The use of specialized culture media, supplemented with growth factors and cytokines, further aids in the expansion and differentiation of cells, such as MSCs or iPSCs, which are key in tissue engineering and regenerative therapies. Recent advances in 3D culture systems and bioreactors have also enhanced the ability to mimic physiological conditions, improving the scalability and therapeutic relevance of cultured cells. These developments are crucial for ensuring the quality and consistency of cell-based products in clinical applications [62,63].

#### 2.3.3. Cell–Cell Interactions

Cell–cell interactions play a crucial role in determining the behavior, function, and fate of cells within regenerative medicine applications. These interactions are mediated by direct contact between cell membranes, such as through gap junctions and cadherin, as well as through secreted signaling molecules, like cytokines and growth factors. Effective cell–cell communication is vital for processes such as differentiation, proliferation, and tissue formation. For example, in tissue engineering, the interaction between MSCs and endothelial cells is essential for promoting vascularization and the development of functional tissues. This dynamic exchange of signals regulates tissue homeostasis and influences how cells integrate into the surrounding microenvironment, making cell–cell interactions pivotal in both natural and engineered tissue formation [64].

In drug delivery systems, understanding and leveraging cell–cell interactions can improve therapeutic outcomes. By designing delivery systems that mimic or enhance natural cell communication pathways, it becomes possible to achieve more targeted and efficient therapies. For instance, nanoparticles and biomaterial scaffolds can be engineered to release bioactive molecules in a stimulus-responsive manner, triggered by specific signals between cells, enhancing tissue regeneration or modulating immune responses. Furthermore, co-culturing different cell types, such as combining stem cells with immune cells, can improve the effectiveness of cell-based therapies by promoting the beneficial interaction between cells within the delivery system, leading to more successful integration and function of the therapeutic cells [65].

#### 2.3.4. Cell Delivery Methods

Effective cell delivery methods are critical for the success of regenerative medicine therapies, as they directly influence the survival, integration, and function of transplanted cells in damaged or diseased tissues. One common approach is direct injection, where cells are delivered via syringe into the target tissue or organ. While this method is simple, it often results in poor cell retention, low survival rates, and uneven distribution. To address these challenges, scaffold-based delivery systems, such as hydrogels and decellularized matrices, have been developed to provide structural support, enhance cell retention, and create a microenvironment that mimics the extracellular matrix. Hydrogels, in particular, offer a tunable platform for cell encapsulation, supporting viability and differentiation. Additionally, microencapsulation within biomimetic hydrogels or biodegradable polymers can protect transplanted cells from immune rejection while enabling controlled release. Injectable biomaterials, such as self-assembling peptides or thermo-responsive gels, further enhance cell survival and integration by forming supportive networks at the target site. These strategies collectively improve the efficacy of cell-based therapies, ensuring more precise and sustained regenerative outcomes [66].

In addition to scaffold-based methods, tissue engineering has advanced with bioactive materials and nanoparticles designed for controlled cell release and signaling to enhance homing, proliferation, and differentiation. For instance, hydrogels and biodegradable polymers, such as alginate and fibrin, serve as cell-laden scaffolds embedded with growth factors like VEGF or BMPs, which are gradually released to promote tissue repair. Moreover, nanoparticles, such as liposomes or polymeric carriers, can simultaneously deliver cells and therapeutic agents, optimizing the microenvironment for regeneration. This integrated approach enhances cell survival, function, and targeted repair, paving the way for more effective and personalized regenerative therapies [67].

#### 2.3.5. Regulatory and Ethical Issues

The use of cell sources in regenerative medicine is subject to complex regulatory and ethical considerations, given the potential for significant impact on human health and well-being. Regulatory bodies such as the U.S. Food and Drug Administration and the European Medicines Agency enforce strict guidelines to ensure the safety, efficacy, and quality of cell-based therapies. These regulations cover everything from the sourcing of cells to manufacturing processes, clinical trial protocols, and post-market surveillance. One key regulatory challenge is the classification of cell-based products as either biologics or medical devices, which determines the pathway for approval. Stem cell therapies, for instance, fall under biologics and must adhere to strict standards for clinical testing before being made available to patients [61]. Additionally, there is a growing emphasis on good manufacturing practices and good clinical practices to ensure that cell therapies are consistently produced and administered with the highest quality standards. Quality parameters include sterility, potency, purity, viability, identity, and stability of the therapeutic cells, as well as rigorous batch-to-batch consistency in manufacturing. Furthermore, preclinical and clinical assessments must demonstrate biocompatibility, controlled differentiation, and long-term safety, reducing risks such as tumorigenicity (ability of cells to form tumors in cancer research) or immune rejection. Meeting these standards is essential for ensuring that regenerative medicine products are both effective and safe for clinical use.

Ethical concerns surrounding cell sourcing, particularly the use of stem cells, add another layer of complexity to regenerative medicine. The debate around the use of ESCs continues, as their obtaining involves the destruction of embryos, raising moral objections for many. In contrast, iPSCs offer a less controversial alternative, as they can be derived from adult cells and reprogrammed to a pluripotent state. Despite this, ethical issues still persist regarding consent, ownership, and the potential for genetic manipulation in cell therapies. Moreover, equitable access to cell-based treatments is a concern, as these therapies often carry high costs and may be available only to select populations. Balancing scientific innovation with public trust in stem cell therapies requires a dual approach: maintaining rigorous regulatory oversight while fostering transparent public engagement. Clear communication about the safety, benefits, and ethical safeguards surrounding ESCs and iPSCs can help build societal confidence. Also, involving stakeholders such as patients, ethicists, and policymakers in decision-making processes supports a more inclusive and ethically responsible advancement of stem cell technologies. Striking a balance between innovation in regenerative medicine and adherence to ethical guidelines is essential to foster public trust and promote equitable access to these life-changing therapies [68].

## 3. Drug Delivery Systems in Regenerative Medicine

### 3.1. Overview of Drug Delivery Approaches

Drug delivery systems play an important role in regenerative medicine by ensuring that therapeutic agents are released at controlled rates and targeted specifically to injured tissues, facilitating enhanced healing. Current approaches use biomaterials such as nanotechnology and polymer-based scaffolds to enable spatiotemporal control, maximizing medication concentrations in the target location while minimizing side effects [69]. For instance, electrospun fiber mats consisting of polycaprolactone (PCL) loaded with nanoparticles that encapsulate growth factors such as Transforming Growth Factor Beta 3 (TGF-β3) have been found to enhance tissue healing by guiding stem cell differentiation in chondrogenic pathways [70]. In addition to linear PLA and PLGA-based systems, branched and star-shaped PLA/PLGA polymers, particularly those modified with polyethylene glycol (PEG), have emerged as promising platforms for drug delivery applications. These architectures enhance drug loading efficiency, stability, and circulation half-life and enable controlled release kinetics with reduced immunogenicity. Such branched structures offer clear advantages over linear analogs, especially in the formulation of nanoparticles, micelles, and hydrogels for controlled and targeted drug delivery. Recent studies have shown that PEG-PLGA copolymers, star-shaped or hyper-branched PLA structures, and PEGylated branched polymers contribute to improved pharmacokinetics and therapeutic outcomes [71,72,73].

Emerging techniques focus on personalized, responsive drug delivery methods, which are especially useful for chronic illnesses that require sustained or long-term release. Mathematical modeling, combined with developments in materials science, has aided in the design of biodegradable and bioresponsive scaffolds, resulting in greatly improved stability, release patterns, and efficacy [69]. Such accuracy in drug delivery has the potential to improve patient outcomes while also enabling effective, long-term regeneration in complex tissue types such as cardiac or nerve tissues [74].

### 3.2. Localized Drug Delivery Techniques

Localized drug delivery techniques play a pivotal role in regenerative medicine by enabling precise, site-specific administration of therapeutic agents, thereby minimizing systemic side effects and enhancing tissue regeneration. These strategies are particularly valuable for creating a favorable microenvironment at the injury site, which is essential for promoting cell survival, proliferation, and differentiation. Techniques such as hydrogel-based carriers, electrospun nanofibers, and microneedles allow for controlled, sustained release of bioactive compounds including growth factors, anti-inflammatory drugs, and antibiotics directly at the site of tissue damage. For example, electrospun nanofibers can be engineered to incorporate nanoparticles that gradually release therapeutic agents, fostering tissue repair while reducing inflammation. These delivery platforms are frequently integrated into biomaterial scaffolds to enhance their regenerative potential [19,75]. Recent advancements in localized delivery systems have introduced more sophisticated technologies, such as 3D-bioprinted constructs and injectable polymer-based carriers, which are designed to closely mimic the mechanical and biochemical properties of native tissues. These systems not only ensure sustained and localized therapeutic release but also contribute to improved vascularization and stem cell proliferation, both of which are critical for tissue regeneration. Moreover, incorporating external stimuli (e.g., magnetic or electrical fields) can further enhance the regenerative efficacy of these delivery systems by triggering on-demand drug release or modulating cell behavior [75].

**Figure 4:**
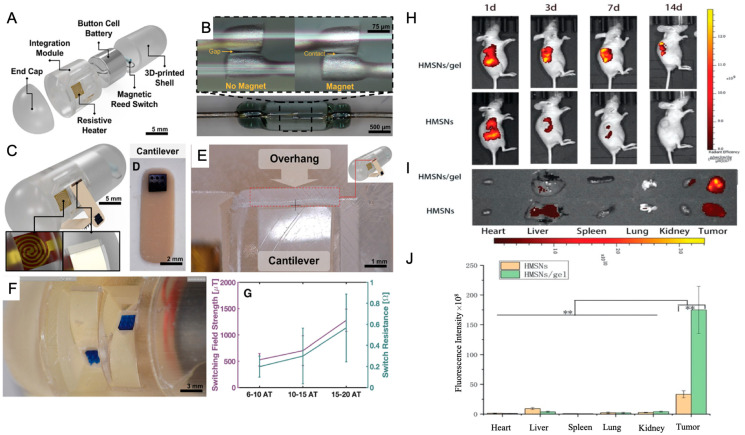
Design overview and magnetic triggering mechanism: (**A**) Computer–aided design showing the system overview, including the electrical and packaging capsule components. (**B**) Close–up of the magnetic reed switch in the open and closed states showing the reed gap and contact, respectively. (**C**) Rendered assembled capsule showing the resistive heating element and the overhang used to restrain the fixed end of the cantilever. (**D**,**E**) The cantilever (**D**) and the cantilever under the overhang (**E**) that restrains during flexure and releases when relaxed. (**F**) Magnified view of two cantilevers before deployment. (**G**) Characterization of the magnetic triggering mechanism showing the switching field strength and switch resistance for different designed switching strengths (n = 3). Data are represented as the mean ± SD. Reprinted and adapted from [76] with permission from Elsevier. In vivo biodistribution and retention were assessed using NIR imaging. (**H**) NIR real–time images of A549 xenograft models were captured after i.t. injection of DiR@HMSNs formulation and DiR@HMSNs/hydrogel composite on days 1, 3, 7, and 14. (**I**) NIR images of ex vivo tumors and mean organs were taken on the 14th day post–injection. (**J**) Quantitative analysis of fluorescence in ex vivo tumors and mean organs was performed. Data are presented as mean ± SD (n = 3), with “**” indicating *p* < 0.01 [77].

Localized delivery approaches are also gaining prominence in organ-specific regenerative medicine. For example, in gastrointestinal (GI) tissue regeneration and disease treatment, innovative oral delivery systems have been developed to overcome the limitations of conventional drug administration, such as low mucosal penetration and off-target effects. One such development is a scalable ingestible capsule that uses a handheld magnet to remotely activate the release of drug-loaded microneedles within the intestinal tract in under 3 s. The microneedles are deployed via a resistive heating mechanism that melts an adhesive, allowing cantilever actuators to inject therapeutics into intestinal tissues. This magnetically triggered system enhances site-specific drug delivery, reduces systemic exposure, and offers improved patient compliance, making it a promising tool for regenerative interventions in the GI tract [76] (Figure 4A–G).

In parallel, localized drug delivery systems are being applied to enhance regenerative cancer therapies. For example, Erlotinib (ERT), a targeted therapy for non-small cell lung cancer (NSCLC), suffers from low oral bioavailability and systemic toxicity. A novel solution involves encapsulating ERT in hollow mesoporous silica nanoparticles (HMSNs) dispersed within a thermosensitive hydrogel matrix composed of PDLLA-PEG-PDLLA (PLEL). This formulation allows for localized, sustained drug release at tumor sites, forming a stable gel upon injection at body temperature. In vivo studies have shown enhanced tumor retention and antitumor efficacy with reduced systemic toxicity, highlighting the potential of such delivery systems in localized and regenerative cancer therapy [77] (Figure 4A–C).

Natural polysaccharide-based materials, such as those derived from dextran and hydroxyethyl starch, have also emerged as effective vehicles for localized antibiotic delivery in regenerative contexts. These hydrogels enable site-specific drug administration, supporting infection control and tissue healing simultaneously. For example, hydrogels containing immobilized antibiotics like amikacin have demonstrated strong antibacterial activity and high biocompatibility, making them suitable for wound healing, bone repair, and scaffold-based tissue engineering applications [78,79,80]. These localized hydrogel platforms are advantageous in maintaining high drug concentrations at the target site while reducing systemic exposure, therefore enhancing therapeutic outcomes in regenerative contexts.

Magnetically responsive drug delivery systems are another emerging approach in regenerative and cancer medicine. Vilas-Boas et al. (2019) [81] demonstrated a dual-population magnetic hyperthermia (MHT) strategy, where non-targeted magnetic nanoparticles (MNPs) are first used to precondition cancer cells, enhancing iron uptake by subsequently administered CXCR4-targeted MNPs. This method significantly improved thermal response during MHT and resulted in complete cell death in glioblastoma (LN229) cells while maintaining minimal cytotoxicity in normal kidney cells (HK-2). Such systems could be adapted to regenerative medicine applications by enabling precise, externally triggered drug delivery that promotes tissue repair or modulates cell activity [81].

**Figure 5:**
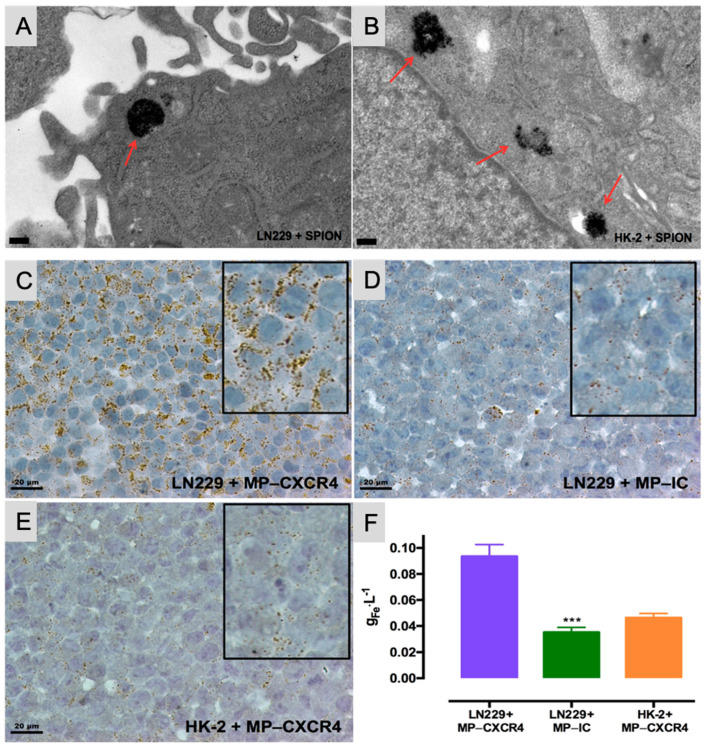
The uptake of SPIONs by LN229 and HK-2 cells is depicted in the transmission electron micrographs. Both LN229 (**A**) and HK-2 (**B**) cells internalize SPIONs, which localize within endosomal compartments (highlighted by red arrows) (scale bars are 200 nm). (**C**–**F**) Interaction of functionalized MPs with LN229 and HK-2 cells. LN229 cells exhibit greater interaction with CXCR4-targeted particles (**C**) compared to IC-functionalized particles (**D**). Additionally, LN229 cells (**C**) show stronger interaction with CXCR4-targeted nanoparticles than HK-2 cells (**E**). Images display reconstituted and stained microtome sections (5 μm thickness; scale bars are 20 μm); magnified details are included as insets. (**F**) Iron quantification by ICP–OES for each condition. Data represent the mean ± SD from two independent experiments. *** *p* < 0.001 compared to LN229+MP-CXCR4 [81].

Figure 5A,B illustrates the uptake of SPIONs by LN229 and HK-2 cells. Transmission electron micrographs reveal that SPIONs localize to endosomal compartments (indicated by red arrows) in both LN229 (A) and HK-2 (B) cells. Figure 5C–F demonstrates the interaction of functionalized MNPs with these cells, showing that LN229 cells engage more extensively with CXCR4-targeted MNPs (C) compared to IC (isotype-control)-functionalized particles (D). Additionally, CXCR4-targeted MNPs interact more with LN229 cells (C) than with HK-2 cells (E). Microtome sections (5 μm thick) were reconstituted and stained; scale bars represent 20 μm, with magnified insets for detail. Panel (F) quantifies iron content for the three experimental conditions using ICP–OES (inductively coupled plasma optical emission spectrometry).

In a related study, Nica et al. (2023) [82] applied a similarly targeted magnetic hyperthermia approach to prostate cancer therapy. By developing trimagnetic core–shell-shell nanoparticles (TMNPs) with high magneto-thermal conversion efficiency, functionalized with prostate cancer cell membranes and cell-penetrating peptides, researchers achieved enhanced cancer cell specificity and apoptosis induction under an external magnetic field. Beyond cancer treatment, this strategy offers broader implications for regenerative medicine, particularly in scenarios requiring targeted activation or modulation of cellular environments to stimulate repair and regeneration [82].

Advances in tissue biofabrication are driving significant progress in regenerative medicine, particularly through the development of three-dimensional (3D) tissue models that better replicate the structural, biochemical, and functional characteristics of native tissues compared to traditional two-dimensional models. These 3D constructs enhance in vitro–in vivo correlation, reduce dependence on animal models, and provide physiologically relevant platforms for studying tissue repair, drug response, and disease progression in regenerative contexts. Among the various biofabrication techniques, bioprinting has emerged as a powerful tool for constructing complex tissue analogs with high spatial and temporal precision.

Bioprinting encompasses various methods, including extrusion-based, droplet-based, and laser-based techniques, each offering distinct advantages for fabricating biomimetic tissues. These technologies enable the precise, layer-by-layer deposition of biomaterials, living cells, and therapeutic agents, facilitating the creation of constructs that closely mimic native tissue architecture. In regenerative medicine, this precision is crucial for orchestrating cell behavior, promoting extracellular matrix (ECM) production, and guiding functional tissue regeneration [83,84].

A key component of bioprinting is the use of bioinks, which are formulated from combinations of cells, polymers, and functional additives. The choice of bioinks plays a critical role in determining the success of tissue regeneration efforts. Scaffold-based approaches, often favored for their practicality and commercial availability, involve immobilizing cells within hydrogels that can support controlled drug release, structural support, and localized micro-environmental modulation. However, they may limit cell migration and long-term viability. In contrast, scaffold-free strategies rely on densely packed cells that self-assemble and secrete their own ECM, better mimicking natural tissue behavior and phenotypic stability. These methods are particularly advantageous for long-term tissue regeneration and modeling. The selection of bioprinting strategies in regenerative applications depends on the specific therapeutic goals and whether the construct is intended to serve as a drug delivery system, a regenerative scaffold, or an in vitro model for tissue repair. Scaffold-based bioprinting is particularly suitable for delivering therapeutic agents in a controlled manner through hydrogel degradation, making it valuable in both tissue engineering and pharmaceutical testing. On the other hand, scaffold-free bioprinting is advantageous for developing fully cellularized tissues that exhibit natural cell–cell interactions and functionality over time [84,85,86,87].

Several studies highlight the growing potential of bioprinting technologies in regenerative applications. For example, Intini et al. (2018) [88] developed a bioprinted scaffold using a 6% chitosan solution enriched with D-(+) raffinose pentahydrate, cooled to −14 °C post-printing using Peltier cells. Gelation was achieved with an 8% *w*/*v* KOH solution, and the constructs were maintained in phosphate-buffered saline (PBS). To enhance regenerative performance, a dense chitosan base layer was incorporated to promote cell retention and tissue ingrowth. When seeded with normal dermal human fibroblasts (Nhdf) and immortal keratinocytes (HaCaT), the constructs supported robust cell proliferation, with significant tissue coverage observed by day 35. The study emphasized the cost-effectiveness, scalability, and biocompatibility of chitosan-based bioprinted scaffolds, particularly for chronic wound healing and skin regeneration [87,88].

**Figure 6:**
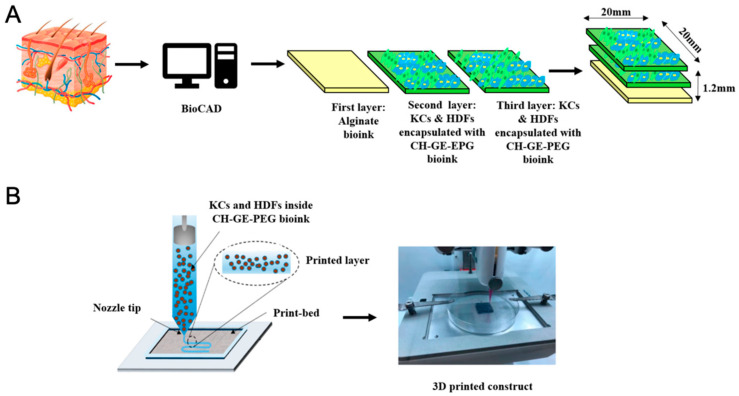
(**A**) Schematic illustration of the design of each layer of skin 3D-printed constructs. (**B**) 3D bioprinting of an alginate layer and KCs and HDFs encapsulated with CH–GE–PEG layers [89].

Similarly, Hafezi et al. (2020) [89] introduced a novel extrusion-based bioprinting strategy utilizing crosslinked chitosan–genipin (CH-GE) bioinks loaded with human dermal fibroblasts and keratinocytes. The optimized CH-GE bioinks demonstrated superior printability, structural integrity, and rheological properties compared to commercial alternatives. With high cell viability (>93%) maintained after seven days, the constructs exhibited favorable macro-porosity for nutrient transport and cell mobility. While initial results confirmed the material’s promise for skin tissue engineering, further enhancements using natural skin-derived biopolymers and longer-term histological studies are needed to achieve fully stratified epidermal and dermal layers [89] (Figure 6). Together, these studies illustrate the growing utility of bioprinting technologies in regenerative medicine, not only for developing functional tissue substitutes but also for facilitating the localized delivery of bioactive agents and supporting natural healing processes. As biofabrication tools continue to evolve, they offer increasingly personalized, reproducible, and scalable solutions to address complex tissue regeneration challenges.

Electrohydrodynamic atomization (EHDA) has emerged as a versatile and transformative technique in regenerative medicine, offering precise control over the fabrication of nano- and micro-scale structures that can be tailored for therapeutic delivery and tissue engineering applications. Among EHDA methods, electrospraying (ES) and electrospinning have become especially prominent due to their ability to produce well-defined fibers with customizable surface properties and encapsulation efficiency. These techniques enable the generation of scaffolds and drug delivery systems that can mimic the extracellular matrix, modulate cell behavior, and support tissue regeneration by delivering bioactive molecules such as growth factors, cytokines, or stem cell-derived factors directly to the repair site. Recent innovations in direct-writing EHDA methods have further enhanced the control and structural precision of deposited materials, overcoming limitations of traditional deposition approaches. These advances not only improve the scalability and reproducibility of biomaterial production but also expand their applicability to cell-topography engineering, localized therapeutic delivery, and functional scaffold design for regenerative therapies. By integrating EHDA-fabricated nanostructures into regenerative platforms, researchers can modulate the local microenvironment, enhance cell adhesion and proliferation, and facilitate controlled drug release, critical elements in accelerating tissue repair and improving clinical outcomes [90,91,92,93,94].

The development of EHDA technologies has also been strengthened by novel engineering strategies, such as multi-tip emitter (MTE) devices and innovative needle geometries. These advancements address challenges like uniform particle generation and scaling production for industrial needs. For instance, MTE devices demonstrated stable atomization at higher flow rates compared to conventional single-needle systems, improving efficiency and particle consistency. Similarly, angled needle designs optimized spray patterns, enhancing the quality and uniformity of nanoparticles. These enhancements highlight EHDA’s potential in producing cost-effective, scalable, and versatile materials for drug delivery, tissue engineering, and nanomedicine, positioning it as a cornerstone of next-generation pharmaceutical technologies [4,95].

Three-dimensional bioprinting has emerged as a pivotal technology in regenerative medicine, enabling the precise, layer-by-layer deposition of bioinks comprising cells, biomaterials, and bioactive molecules in a scaffold-free or scaffold-based manner to closely mimic the architecture and functionality of native tissues. This technique facilitates the fabrication of biomimetic, scalable constructs with complex geometries and spatial heterogeneity, advancing beyond the structure and functional limitations of conventional scaffold-based methods. In regenerative applications, bioprinted tissues can be pre-matured in vitro within bioreactors to support cellular differentiation and matrix development prior to implantation or directly printed in situ, utilizing the body as a natural bioreactor to guide tissue formation and integration. The biomedical relevance of 3D bioprinting lies in its ability to generate structurally and functionally relevant tissue models for bone, cartilage, skin, vascular structures, and even patient-specific constructs such as human-scale ear cartilage, supporting both tissue repair and therapeutic delivery. Moreover, this technology is increasingly applied in localized drug delivery systems, where bioprinted matrices are engineered to provide sustained, site-specific release of therapeutic agents such as growth factors or small-molecule drugs, further enhancing tissue regeneration outcomes. A range of bioprinting modalities, including extrusion-based printing, inkjet printing, laser-assisted bioprinting, and cell electrospinning, offer distinct advantages based on cell type, resolution, and intended application. These strategies not only improve cell viability and spatial distribution but also enable the customized design of tissue-specific microenvironments, crucial for promoting cellular interactions, matrix deposition, and functional tissue development [96,97].

**Figure 7:**
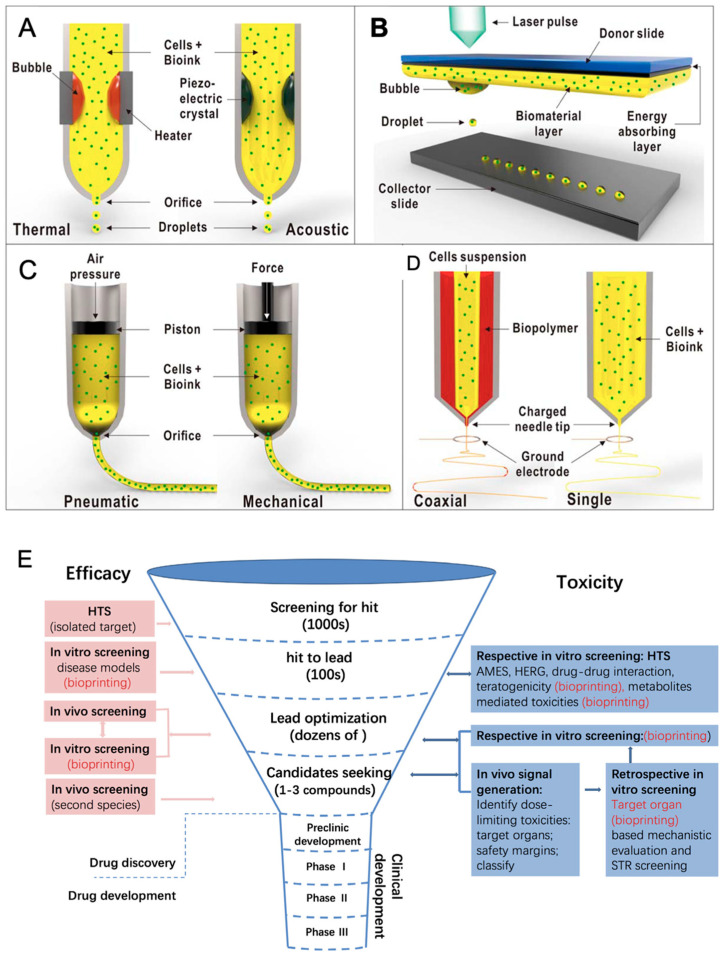
Different types of bioprinting techniques for fabricating cell-laden constructs: (**A**) Inkjet bioprinting utilizes thermal heaters or piezoelectric crystals to create bubbles that expel bioink droplets. (**B**) Laser-assisted bioprinting uses laser light to generate bubbles in a biomaterial layer in the ribbon (donor slide/energy absorbing layer). (**C**) Extrusion bioprinting employs a syringe with a piston driven by air pressure or mechanical force to release bioinks through the orifice in a continuous stream or droplets depending on the viscosity of the material. (**D**) Bioelectrospraying/cell electrospinning uses a charged needle tip at the orifice to accelerate and control the expulsion of bioinks. Reprinted with permission from reference [100]. Copyright (2017) John Wiley & Sons, Inc. (**E**) Applications of bioprinting in drug discovery and development: developmental toxicity screening, cytotoxicity testing with organoids, structure-toxicity studies, and enhanced in vitro efficacy screening using bioprinted disease models. Reprinted from reference [83] with permission from Elsevier.

Despite the rapid advancements, there remains a notable gap in comparative studies evaluating bioprinting methods and bioinks for specific pharmaceutical applications. This lack of standardized benchmarks makes it challenging for researchers to determine the most appropriate printing techniques and materials for particular therapeutic needs. Comparative investigations are essential to assess parameters such as print fidelity, cell viability, mechanical properties, and biological functionality across different printing modalities and bioink compositions. Addressing this gap would greatly support method selection, protocol optimization, and translational research in pharmaceutical development [98,99]. This field continues to push the boundaries of regenerative medicine and biomedical innovation [100] (Figure 7). Furthermore, bioprinting has emerged as a transformative technology in the drug discovery and development process (preclinical phase), offering advanced tools to model biological systems more accurately. One key application is teratogenic or developmental toxicity screening, where bioprinted systems enable the differentiation of stem cells within scaffolds to predict developmental toxicity. Additionally, bioprinted human metabolism organoids facilitate cytotoxicity testing by producing active metabolites that improve the prediction of in vivo toxicities. Beyond toxicity assessment, bioprinted organoids allow for mechanical evaluations and the study of structure-toxicity relationships, helping to identify safer and more effective drug candidates. Also, bioprinted disease models with human cells enhance the reliability of in vitro efficacy screening, bridging the gap to in vivo outcomes. These diverse applications are schematically illustrated in Figure 7E [83].

**Table 1:** Overview of cellular components in regenerative medicine.

Cell Type	Key Features	Applications in Regenerative Medicine	Integration with Drug Delivery Systems	Ref.
Stem Cells				
Embryonic Stem Cells (ESCs)	Pluripotent; potential for infinite growth; Derived from the embryonic inner cell mass	Spinal injury, cardiovascular diseases, and neurodegenerative disorders; Limited due to ethical and political concerns	Controlled-release systems for protecting and sustaining growth factors; Smart scaffolds for directed differentiation	[101,102]
Adult Stem Cells	Undifferentiated, found in most organs; Capable of tissue regeneration and self-renewal	Treat degenerative disorders and cancers; Effective in bone marrow and skin grafts	Bioactive scaffolds supporting tissue repair; Nanoparticles for precise delivery of therapeutic agents	[103,104]
Induced Pluripotent Stem Cells (iPSCs)	Reprogrammed adult cells resembling ESCs; Personalized, patient-specific	Used in disease modeling, drug screening, and patient-specific therapies	Biomaterials for controlled differentiation Smart patches delivering bioactive molecules	[59,61]
Progenitor Cells	Limited self-renewal and differentiation potential; Involved in tissue repair	Targeted tissue regeneration in neurodegenerative diseases, heart disease, and diabetes	Sustained release of differentiation factors via nanoparticles; Scaffolds mimicking native environments	[105]
Somatic Cells	Specialized non-reproductive cells; Replace and repair damaged cells	Tissue engineering for skin, liver, muscle, and nerve regeneration	Drug-loaded liposomes targeting specific cell types; Regenerative skin patches	[46,47]
Immune Cells	T cells: adaptive immunity, CAR-T therapies; Dendritic cells: antigen presentation	Cancer immunotherapy, vaccine development, and regenerative immunology	Nanoparticles enhancing antigen presentation; Engineered delivery vehicles targeting immune cells	[50]
Endothelial Cells	Line blood vessels; critical for angiogenesis	Vascularization in tissue engineering and wound healing	Targeted drug delivery for vascular diseases; Nanoparticles exploiting tumor vasculature for drug delivery	[53,54,55]
Chondrocytes	Maintain cartilage extracellular matrix	Cartilage regeneration and repair	Hydrogels for sustained release of anti-inflammatory agents; Scaffolds delivering growth factors	[56]
Osteoblasts	Bone formation and mineralization	Bone repair and regeneration	Scaffolds loaded with BMPs for accelerated healing; Controlled release of osteogenic agents	[57]
Myocytes	Muscle cells responsible for contraction	Muscle regeneration for degenerative diseases and injuries	Hydrogels releasing IGF-1 for enhanced regeneration; Nanoparticles delivering bioactive molecules	[58]

### 3.3. Controlled Release Systems

A drug, or active pharmaceutical ingredient (API), is a substance recognized in official pharmacopeias and intended for use in diagnosing, curing, mitigating, treating, or preventing disease, as defined by the FDA. Drug delivery refers to techniques that enhance drug concentration at specific target sites in the body, optimizing efficacy and reducing systemic exposure. The primary objective of any delivery system is to ensure targeted, extended, and protective drug interaction within diseased tissues. Dosage forms combine APIs, the chemical agents treating diseases, with excipients or additives, which are non-drug components. APIs are rarely administered alone due to challenges such as difficulty in accurate dosing for potent drugs, degradation in hostile environments like stomach acid, local irritation, or instability caused by environmental factors. APIs may also have unpleasant sensory properties like taste or smell, reducing patient compliance. Excipients address these issues by stabilizing formulations, masking unpleasant qualities, enabling precise dosing, and facilitating manufacturing. They also improve bioavailability, enhance safety, and make the formulation more acceptable and functional during storage or use, in due course improving patient adherence [1,106].

**Figure 8:**
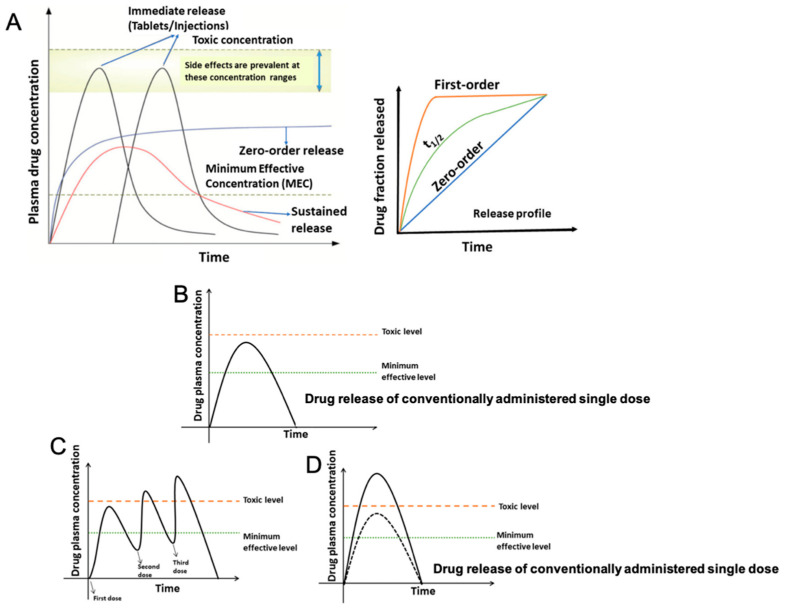
Drug release profile illustrating plasma-drug concentration over time, with indicated thresholds for minimum effective and toxic concentrations. Maintaining drug levels within this therapeutic window is essential for efficacy and safety: (**A**) Drug plasma levels and release profiles. Plasma drug concentration over time following the administration of (**B**) a single conventional dose, (**C**) multiple doses, and (**D**) an increased single dose [1].

The drug release profile is typically represented as a graph plotting plasma drug concentration against time. As shown in Figure 8A, two key concentration thresholds are highlighted: the minimum effective concentration, below which the drug produces no therapeutic effect, and the toxic concentration, above which harmful side effects may occur. Maintaining the drug concentration within this therapeutic window, between these two levels, is essential for ensuring both safety and effectiveness. Drug release follows zero-order kinetics when the drug is eliminated at a constant rate, regardless of its concentration. Zero-order drug delivery systems address the limitations of immediate-release and first-order systems by providing a steady release of the drug, ensuring consistent plasma levels within the therapeutic window over an extended duration. Conventional drug delivery systems (DDSs), such as tablets, capsules, and syrups, are rapidly eliminated from the body, making it difficult to maintain drug concentrations within the therapeutic window. After administering a single conventional dose, the drug level in the plasma rises quickly but declines exponentially shortly after, often failing to sustain a significant therapeutic effect and leading to sub-therapeutic responses. Figure 8B illustrates the fluctuations in plasma drug levels with conventional DDSs. To maintain plasma drug concentrations between the minimum effective concentration and the toxic concentration, various approaches have been explored. Administering multiple doses at regular intervals may seem like a solution, but it causes significant plasma level fluctuations, often dipping below effective levels or exceeding toxic levels. Furthermore, taking several doses throughout the day leads to poor patient compliance. Another strategy involves administering a single, larger dose, which can result in adverse effects due to excessive drug levels. Controlled release DDSs are therefore essential, as they maintain plasma drug concentrations at a steady rate within the therapeutic window, ensuring prolonged therapeutic efficacy and improved patient outcomes [1,106].

Moreover, conventional DDSs offer several advantages, including convenient and non-invasive administration, accurate and measured dosing, better in vitro–in vivo correlation (IVIVC), longer shelf life, flexibility for dose adjustments by physicians, and low cost. However, they also have significant drawbacks, such as poor absorption at the site of administration, lack of target specificity, premature drug metabolism or excretion, low bioavailability, the need for frequent dosing, and poor patient compliance. In contrast, controlled DDSs provide benefits like precise and sustained drug release, target specificity, prolonged drug residence time, protection from enzymatic or chemical degradation, improved bioavailability, reduced dosing frequency, and better patient compliance. Despite these advantages, controlled DDSs have limitations, including the potential toxicity of materials used, risks of dose dumping, invasive procedures for implantation or removal, reduced efficacy due to uptake by the reticuloendothelial system (RES), weaker IVIVC, limited standards, and higher manufacturing costs [1,106].

Furthermore, carrier-free, noncovalent nanoparticles (NPs) based on natural products have shown promising potential in enhancing chemo-photodynamic combination therapy for cancer treatment. These smart NPs offer advantages such as high biocompatibility, improved pharmacological activity, and enhanced therapeutic efficacy compared to free photosensitizers and conventional drugs. Recent studies have demonstrated their synergistic anticancer effects and highlighted their role as a transformative approach in improving photodynamic therapy outcomes through improved spatiotemporal precision and reduced invasiveness [107]. Also, recent advancements in drug delivery have shifted from traditional carrier systems to the development of “smart” micro- or nanocarriers with tailored properties such as size, shape, surface charge, and biodegradability, enabling improved drug loading, release profiles, and cellular uptake. The combination of stimulus-responsive components has led to multifunctional carriers that not only deliver drugs efficiently but also exert therapeutic effects themselves, such as reactive oxygen species modulation, immunomodulation, or anti-inflammatory actions, enhancing the overall therapeutic efficacy [108].

Moreover, polysaccharide-based hydrogels, particularly those derived from dextran and hydroxyethyl starch, have shown considerable potential as controlled release platforms for antibiotics in regenerative medicine. These hydrogels enable a sustained and gradual release of immobilized drugs like amikacin, offering prolonged antimicrobial protection while supporting tissue regeneration processes [78,79,80]. The release kinetics can be tailored by modifying crosslinking density and polymer composition, allowing precise control over drug diffusion rates and therapeutic duration. These systems represent an important intersection between antibacterial functionality and regenerative support and broaden the design landscape for multifunctional drug delivery platforms.

**Figure 9:**
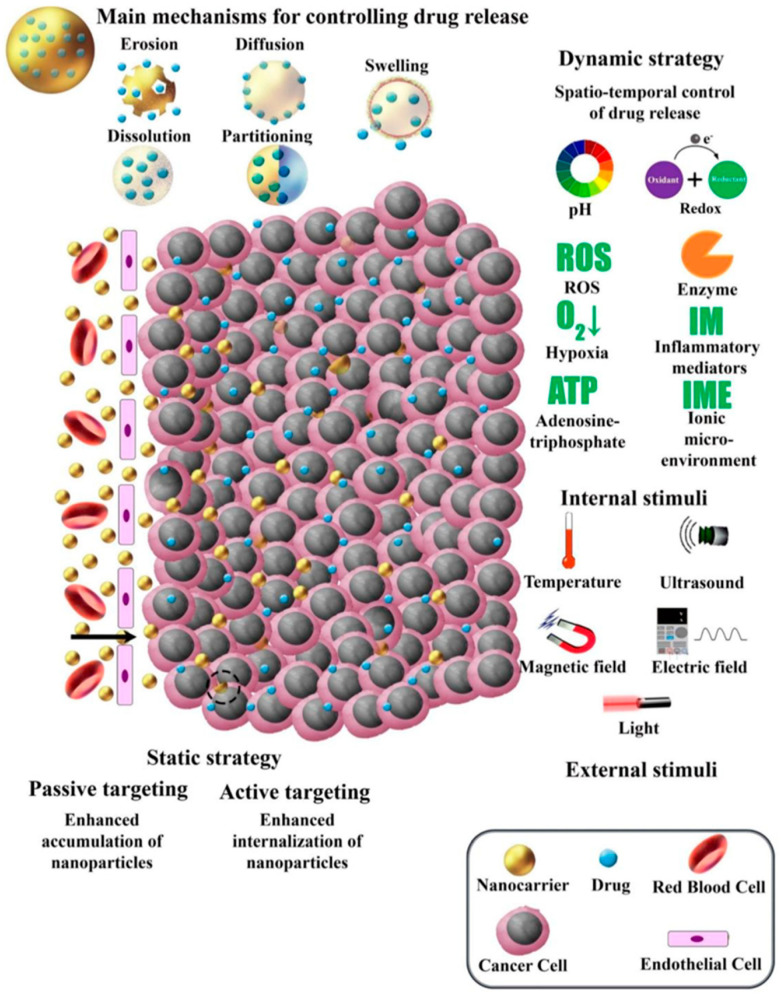
Key mechanisms of drug release from nanocarriers and contemporary nano-based drug delivery systems for cancer therapy. Reprinted from reference [106] with permission from Elsevier.

The common approach to drug delivery involves injecting nanoparticles intravenously, allowing them to interact with blood vessel endothelium and selectively enter the tumor interstitium. Once inside, the nanoparticles must be absorbed by cancer cells and retained long enough to achieve their therapeutic effect. DDSs address the challenges of targeting by using two main strategies. The static targeting strategy includes passive targeting, based on the enhanced permeation and retention (EPR) effect, and active targeting, where nanocarriers are modified with ligands to bind cancer cell receptors for internalization. The dynamic targeting strategy provides an alternative, using internal or external stimuli to release drugs from carriers, offering flexibility in drug conductance and release profiles (Figure 9). For a DDS to be effective, these strategies must be carefully optimized, and the appropriate drug release method selected to suit the therapeutic goal.

Furthermore, tumor-targeted nanomaterial-based drug delivery systems have emerged as a highly effective approach for cancer treatment due to their stability in blood circulation, predictable delivery patterns, enhanced tumor-selective drug accumulation, and reduced toxicity to healthy tissues. The cell-surface glycoprotein CD44, which binds to the extracellular domain of hyaluronic acid (HA), is overexpressed in various cancers, including breast, ovarian, lung, and stomach cancers. Yu, T. et al. (2020) [109] developed an HA-based nano-carrier loaded with doxorubicin (DOX) and cisplatin (CDDP) as a CD44–targeted drug delivery system and evaluated its tumor-suppressive effects on CD44^+^ breast cancer cells both in vitro and in vivo. The dual-drug-loaded HA micelles (HA-DOX-CDDP) demonstrated pH-sensitive drug release, taking advantage of the acidic tumor microenvironment.

In acidic conditions (such as those found in tumor tissues), the ionization of HA weakens its interaction with the loaded drugs, leading to faster drug release and improved therapeutic efficacy.

This selective drug activation minimizes systemic toxicity while maximizing tumor-specific treatment. Additionally, HA-DOX-CDDP micelles showed higher cellular uptake and stronger growth inhibition compared to free drugs in 4T1 (CD44^+^) breast cancer cells, while no significant differences were observed in NIH-3T3 (CD44^−^) control cells. In a 4T1 mammary cancer mouse model, HA-DOX-CDDP micelles exhibited greater tumor suppression and reduced systemic toxicity than free drugs, as confirmed by immunofluorescence and histological analyses. These findings highlight that HA-DOX-CDDP micelles integrate acid-sensitive drug release, CD44-targeted delivery, and excellent biocompatibility and biodegradability, making them a promising platform for enhanced chemotherapy in breast cancer [109].

#### Controlled Release Systems: Mathematical Models, Equations, and Applications

Controlled release systems are designed to deliver drugs in a sustained, predictable manner, optimizing therapeutic efficacy while minimizing side effects. Mathematical models play a crucial role in understanding and predicting drug release kinetics from nanocarriers and other delivery platforms. These models help in designing formulations that achieve desired release profiles by accounting for factors such as diffusion, degradation, and matrix erosion. Below, key models are outlined along with their descriptions, governing equations, and typical applications:

I. Diffusion-Based Models

Diffusion-based models are foundational in understanding drug release mechanisms. These models describe how drugs migrate from a delivery system, such as a polymer matrix or hydrogel, into surrounding tissues or fluids. This movement is governed by Fick’s laws of diffusion, where the driving force is a concentration gradient. Diffusion-based models are widely applicable to systems where the drug is released passively over time without significant involvement of carrier degradation or swelling. Diffusion-based models are commonly used in hydrogels, microneedles, and matrix tablets to predict the passive, sustained release of small molecules or biologics and are stated by the following Equation (1) [106,110]:

(1) $$J=-D\frac{dc}{dx}$$

where

J = diffusion flux (amount of drug diffused per unit area per unit time);

D = diffusion coefficient (material-dependent constant);

dc/dx = concentration gradient of the drug.

II. Higuchi Model

The Higuchi model is one of the earliest and most widely used mathematical models for drug release from matrix systems. It assumes that drug release is controlled by diffusion through a homogeneous polymer matrix. This model is based on the premise that the drug is initially uniformly distributed throughout the matrix, and its release rate decreases over time due to the square-root dependency. The Higuchi model is particularly useful for transdermal patches, controlled-release implants, and certain topical drug formulations and is represented by the following Equation (2) [111]:

(2) $$Q=k_{H}\sqrt{t}$$

where

Q = cumulative amount of drug released;

k_H_ = Higuchi constant (dependent on drug solubility and matrix properties);

t = time.

III. Peppas (Korsmeyer–Peppas) Model

The Peppas model is a versatile tool for analyzing drug release from polymeric systems. It accounts for both Fickian diffusion and non-Fickian mechanisms such as polymer swelling and degradation. The release mechanism is characterized by the release exponent (n), which helps distinguish between diffusion- and erosion-controlled release. For this reason, the Peppas model is especially valuable in evaluating complex systems. This model is frequently applied to hydrogels, nanoparticles, and biodegradable polymers, especially when drug release is influenced by a combination of swelling, erosion, and diffusion mechanisms and expressed as follows in Equation (3) [111,112]:

(3) $$M_{t}/M\infty \;=k_{P}t^{n}$$

where

M_t_/M∞ = fraction of drug released at time *t*;

k_P_ = Peppas release rate constant;

t = time;

n = release exponent (indicative of the mechanism).

IV. First-Order Kinetics

First-order kinetics describe systems where the release rate is proportional to the drug concentration remaining in the delivery system. This means that as the drug depletes, the release rate slows down over time. First-order kinetics are common for systems where the drug dissolves or diffuses uniformly and rapidly into the surrounding environment. This model is widely applicable in immediate-release tablets, capsules, and some biodegradable microparticles and is represented by the following Equation (4) [113]:

(4) $$\mathrm{Log}\;C=\mathrm{Log}\;C_{0}-k_{t}/2.303$$

where

C = drug concentration;

C_0_ = the initial drug concentration;

k = the first-order rate constant;

t = time.

The release data, represented as the percentage of the remaining drug over time, form a straight line with a slope of k/2.303.

V. Zero-Order Kinetics

Zero-order kinetics describe drug delivery systems that release drugs at a constant rate over time, regardless of drug concentration. This type of release profile is ideal for maintaining a steady drug level in the body, which is particularly important for chronic diseases where constant therapeutic levels are required. Zero-order kinetics are frequently observed in osmotic pumps, transdermal patches, and certain types of controlled-release implants and expressed as follows in Equation (5) [114]:

D_t_ = D_0_ + k_0_t (5)

where

D_t_ = the amount of drug dissolved at time t;

D_0_ = the initial drug amount in the solution;

K_0_ = the zero-order release rate constant.

VI. Weibull Model

The Weibull model is a flexible, empirical approach for fitting drug release data to various release patterns, including both controlled-release and immediate-release systems. By adjusting its shape and scale parameters, the model can describe different drug release profiles over time, ranging from fast to slow-release kinetics. This model is used for fitting experimental data in controlled-release formulations, including polymer systems and oral dosage forms, as well as both immediate and sustained drug-release patterns using the following Equation (6) [106]. Furthermore, the effective surface area, which depends only on mass, is a factor affecting overall drug release.

M = M_0_ [1 − exp [(t − T)^b^/a)] (6)

where

M = the amount of drug dissolved;

M_0_ = total amount of drug being released;

T = the lag time measured as a result of the dissolution process;

b = shape parameter (defines the release pattern);

a = scale parameter (defines the time scale of release).

VII. Hixson-Crowell Model

The Hixson-Crowell model is used to describe drug release controlled by surface erosion or dissolution, where the size of the delivery system changes over time. It assumes that the drug release rate is proportional to the surface area of the degrading particle or matrix. This model is typically applied to systems governed by surface erosion mechanisms, such as biodegradable implants or solid particles, and is expressed by the following Equation (7) [106,115]:

W_0_^1/3^ − W_t_^1/3^ = k_s_t (7)

where

W_0_ = initial amount of drug;

W_t_ = drug mass remaining at time t;

K_s_ = Hixson-Crowell release constant.

VIII. Hopfenberg Model

This model describes drug release from surface-eroding polymer systems, considering geometry factors like slabs, spheres, or cylinders. It is particularly useful for evaluating systems where drug release is proportional to the surface area of the degrading carrier. The Hopfenberg model is often used in erosion-controlled polymeric drug delivery systems, such as implants or coatings, which have degradable surfaces with consistent degradation rates, as described by the following Equation (8) [116]:

M_t_/M_∞_ = 1 − [1 − (k_0_t/C_0_a)]^n^ (8)

where

M_t_ = the drug release amount at t;

M_∞_ = the amount of drug released at infinite time;

k_0_ = the erosion rate constant;

C_0_ = the initial concentration of drug in the system;

a = the radius or half thickness;

n = the geometry factor depends on the shape, with values of 1 for a thin film, 2 for a cylinder, and 3 for a sphere.

IX. Sequential Layer Model

The Sequential Layer Model is designed for drug delivery systems that release drugs in a stepwise manner, often due to the degradation or dissolution of distinct layers. Each layer contains a specific drug or dose, and release occurs sequentially as the layers degrade or dissolve over time. This model is particularly relevant in multi-drug systems or layered drug delivery platforms. This model is commonly applied in cancer therapies requiring sequential drug release, layered wound healing scaffolds, and combination therapies where precise timing of drug release is critical and expressed by the following Equation (9) [106,117,118]:

(9) $$M_{\mathrm{pt}}=M_{\mathrm{po}}-k_{\mathrm{diss}}A_{t}$$

where

M_pt_ = mass of drug remaining at time t;

M_po_ = initial mass of drug;

k_diss_ = dissolution rate constant of the layer;

A = surface area of the layer.

## 4. Challenges and Limitations

### 4.1. Biological Barriers

Biological barriers present significant challenges in advancing drug delivery systems for regenerative medicine. The human body’s natural defense mechanisms hinder the efficient transport of therapeutic agents to target sites. Primary barriers such as the skin, mucus, and mucosal layers prevent external substances from entering the body. Secondary barriers, including the blood–brain barrier and endothelial layers, regulate internal molecular transport, restricting drug penetration into critical tissues. At the tissue level, the ECM serves as a tertiary barrier, impeding drug diffusion through its dense, interconnected network. These barriers collectively limit the bioavailability and efficacy of therapeutic agents while increasing the risk of off-target effects, emphasizing the need for innovative strategies to enhance targeted drug delivery.

Moreover, the heterogeneity of biological barriers across different tissues complicates the design of universal drug delivery systems. Each tissue type, such as bone, cartilage, or neural tissue, has its unique structural and functional characteristics that influence drug permeability. Recent advances in nanotechnology, such as the development of nanoparticles and nanocarriers, aim to overcome these barriers, but ensuring their efficient targeting and minimal off-target effects remains a significant hurdle. For example, the use of biomimetic nanoparticles designed to mimic the surface properties of specific cells may improve targeted delivery, but their ability to navigate complex tissue environments still needs optimization. Therefore, understanding and overcoming biological barriers remains crucial for the successful integration of drug delivery with regenerative medicine [119,120].

### 4.2. Safety Concerns in Drug Delivery Systems

Safety remains a critical concern in the development of drug delivery systems, particularly those integrated with regenerative medicine. While these therapies offer substantial clinical benefits, they also pose risks such as immunogenicity, toxicity, and off-target effects. One major challenge is immune system activation, which can lead to chronic inflammation, rejection, or systemic toxicity. Gene therapies and stem cell-based treatments, for example, may provoke immune responses against foreign biological materials, leading to adverse effects. Additionally, prolonged exposure to drug carriers, such as liposomes or nanoparticles, may trigger inflammatory reactions or interfere with normal physiological processes. The biocompatibility and biodegradability of materials used in drug delivery are key safety considerations. While some materials, such as PEG and chitosan, are considered biocompatible, concerns remain about their accumulation and clearance from the body. Drug carriers that are not efficiently metabolized may pose long-term safety risks, including organ toxicity. Furthermore, systemic toxicity can arise when drug carriers release therapeutic agents unintentionally into non-targeted tissues, affecting healthy organs.

To address these safety challenges, optimizing drug delivery systems through improved targeting specificity, enhanced biodegradability, and reduced immunogenicity is essential for their safe and effective application in regenerative medicine. Table 2 summarizes commonly used materials for drug carriers, their toxicity profiles, and metabolic pathways for degradation [121,122].

### 4.3. Regulatory Hurdles

The integration of drug delivery systems with regenerative medicine faces significant regulatory challenges due to the complexity of these therapies and the evolving landscape of regulatory frameworks. Regulatory agencies such as the FDA and EMA have yet to establish comprehensive guidelines specific to combination therapies involving regenerative medicine and drug delivery technologies. Unlike traditional pharmaceutical products, regenerative therapies often involve living cells, biologics, and complex materials, which require extensive preclinical and clinical testing. This results in longer timelines for approval and increased costs. Also, the need for rigorous post-market surveillance and personalized treatment approaches introduces additional regulatory complexity.

Moreover, the regulatory uncertainty surrounding new technologies such as gene editing or stem cell therapies complicates the approval process for drug delivery systems. The risk of unintended genetic modifications or the potential for tumorigenesis in certain stem cell therapies is a major concern for regulators. To ensure consistency in manufacturing processes, controlling product variability, and developing biomarkers for efficacy and safety remain key issues in regulatory approval. To address these challenges, there is a growing need for collaboration between researchers, clinicians, and regulatory bodies to create clear guidelines and facilitate the transition of innovative therapies from the laboratory to the clinic [131,132].

## 5. Future Directions and Emerging Technologies

The integration of advanced drug delivery systems with regenerative medicine is poised to drive transformative progress, particularly within the framework of personalized medicine. As highlighted in Section 3, the ability to spatially and temporally control therapeutic release is pivotal for enhancing treatment efficacy while minimizing systemic side effects. Emerging technologies such as 3D bioprinting, microfluidics, and nanotechnology-based systems are redefining this landscape by enabling the fabrication of patient-specific constructs and smart delivery platforms. These innovations support precision-targeted therapies through responsive materials that adapt to physiological cues, improving outcomes in tissue regeneration, cancer therapy, and localized treatment of chronic diseases. Furthermore, convergence with digital health technologies, such as AI-guided design of personalized drug delivery systems and organ-on-chip models, offers promising avenues for real-time treatment optimization and high-throughput drug screening. As these technologies continue to evolve, their integration into clinical workflows will facilitate tailored, adaptive, and more effective regenerative treatments, ultimately bridging current translational gaps in biomedicine.

### 3D Bioprinting and Personalized Scaffolds

As mentioned above, 3D bioprinting has opened new horizons in regenerative medicine by enabling the fabrication of patient-specific scaffolds that serve dual functions as drug delivery systems and as structural matrices for tissue regeneration. Utilizing patient-derived cells and biocompatible bioinks, these scaffolds can be precisely engineered to replicate the architectures and microenvironment of native tissues. Moreover, they can be functionalized with bioactive molecules such as growth factors, cytokines, and small-molecule drugs, allowing spatiotemporally controlled therapeutic release to enhance regeneration efficacy. Future directions in this field are expected to focus on integrating real-time imaging, computational modeling, and artificial intelligence (AI) to refine scaffold design, predict biological responses, and improve clinical outcomes [133,134,135].

Currently, 3D bioprinting is gaining increasing attention in bone and cartilage regeneration. In bone tissue engineering, bioprinted scaffolds are designed not only to provide mechanical support but also to foster key cellular processes, including cell adhesion, migration, proliferation, and differentiation. Among various biomaterials, poly(lactide) (PLA) has emerged as a leading candidate due to its favorable biocompatibility, biodegradability, and printability, making it suitable for constructing functional bone substitutes [136]. Researchers have explored the combination of 3D-printed PLA scaffolds with human gingival mesenchymal stem cells (hGMSCs) and extracellular vesicles (EVs) to evaluate their cytotoxicity and regenerative potential. Importantly, the degradation byproducts of PLA did not elicit any cytotoxic response. In an in vivo study, rats with cortical calvaria bone defects showed the formation of new bone nodules and blood vessels in the calvariae after six weeks of implantation with these scaffolds [137,138]. Furthermore, Teixeira et al. (2019) [139] demonstrated that the osteoinductive properties of 3D-printed PLA scaffolds could be enhanced by incorporating polydopamine (PDA) and type-I collagen as surface coatings. This modification further underscores the potential of PLA-based scaffolds to support bone regeneration and vascularization in tissue engineering applications [139]. Recent studies have demonstrated that dopamine-functionalized polymers, synthesized via efficient click reactions with epoxy-containing polymers, offer enhanced adhesive properties and surface modification potential while enabling more controllable and scalable production methods [140]. Additionally, dopamine-containing hydrogels prepared through immobilization techniques have shown promising properties, such as pH sensitivity, controlled degradation, and suitability for biomedical applications [141]. These materials represent a valuable addition to the toolbox of biofunctional platforms in regenerative medicine and drug delivery systems.

**Figure 10:**
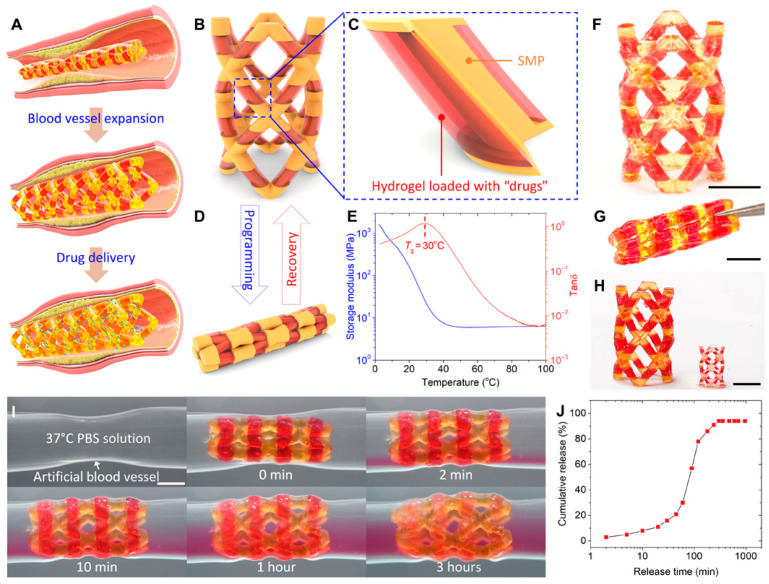
Printed SMP stent with drug–releasing function: (**A**) Illustration of the stent’s blood vessel expansion and drug–releasing functions. (**B**) Overall design of the SMP–hydrogel stent. (**C**) Detailed design showing SMP rods surrounded by drug–loaded hydrogel skins. (**D**) Compact shape programming of the SMP–hydrogel stent. (**E**) DMA results indicate the SMP’s Tg is 30 °C. (**F**–**H**) Snapshots of the SMP–hydrogel stent: (**F**) as–printed, (**G**) compacted shape, and (**H**) stents of varying sizes. (**I**) Demonstration of shape memory and drug-releasing functions. (**J**) Quantified drug release process (scale bar: 5 mm) (photo credit: Jianxiang Cheng, Southern University of Science and Technology) [142].

Hydrogel-polymer hybrid systems hold considerable promise for biomedical device development due to their combined mechanical robustness and biological compatibility. However, traditional fabrication methods typically constrain these hybrids to simple laminate geometries, often limiting their functional integration and application potential. Recent advancements in multi-material 3D printing have enabled the creation of complex, covalently bonded hydrogel-polymer architectures. Specifically, a novel approach utilizing a custom digital light processing (DLP)-based printer allows precise fabrication of highly stretchable, water-rich acrylamide–poly(ethylene glycol) diacrylate (PEGDA) hydrogels in combination with UV-curable elastomeric polymers. This technique facilitates the seamless integration of disparate materials within a single construct, significantly expanding the functional design space of hydrogel-polymer systems. As a representative application, shape memory polymer (SMP)-based stents with embedded drug-delivery capabilities have been successfully fabricated, demonstrating the potential of this strategy for developing multifunctional, stimuli-responsive biomedical devices. As shown in Figure 10A, SMP is an ideal material for 4D printing stents that expand narrowed blood vessels. By integrating hydrogel into SMP rods using multi-material 3D printing, drug-releasing functionality is achieved (Figure 10B,C). The stent’s shape memory effect allows it to be compacted at a programming temperature, fixed below its glass transition temperature (Tg), and recover to its original shape when reheated (Figure 10D). Modified UV polymer (VeroClear) lowers the Tg to 30 °C for effective programming at 37 °C and compacting at 20 °C (Figure 10E).

Using a custom multi-material DLP printer, SMP-hydrogel stents are fabricated with red dye as a model drug (Figure 10F,G). This approach enables size customization for different blood vessels (Figure 10H). Ge et al. (2021) [142] demonstrated the stent’s shape memory and drug-releasing functions in a simulated stenotic vessel (Figure 10I). Upon insertion, the stent expands within 2 min and fully opens in 1 h, while the hydrogel releases drugs, quantified using UV-visible spectroscopy (Figure 10J). Drug release is adjustable by tuning the hydrogel’s mesh size, using hydrogel-particle mechanisms, or adopting environmentally responsive hydrogels that react to pH or temperature changes [142,143,144].

Cardiovascular diseases remain the leading cause of mortality in the United States, yet cardiovascular drugs continue to exhibit clinical trial failure rates of up to 80%. This alarming statistic highlights a pressing need for innovation in cardiovascular drug discovery and development. High attrition and post-market withdrawal rates are largely attributed to the lack of physiologically relevant 3D microenvironments in traditional cell culture models used for cardiotoxicity screening, as well as variability in drug responses across diverse populations (e.g., different ethnic groups, older adults). These challenges underscore the critical demand for more representative and personalized biomimetic screening platforms [145,146].

Emerging technologies such as 3D bioprinting offer transformative solutions by enabling the fabrication of advanced cardiac tissue models that better replicate the structural and functional characteristics of native myocardium. Notably, recent studies demonstrated that a 3D-bioprinted micro-physiological device exhibited inotropic responses to verapamil, an L-type calcium channel blocker, that closely mirrored those observed in isolated whole postnatal rat hearts. These promising results emphasize the utility of bioprinted cardiac constructs as physiologically relevant platforms for preclinical drug testing. Continued progress in engineered cardiac tissues, combined with precision medicine approaches, positions 3D bioprinting as a pivotal tool for reducing attrition rates and enhancing the safety and efficacy of cardiovascular therapeutics [133,147,148,149,150].

For example, chitosan, a naturally derived polysaccharide, is well-regarded for its biocompatibility, biodegradability, and ability to form porous matrices, making it a versatile material for drug delivery applications. When integrated with 3D printing technologies, chitosan-based bioinks can be used to fabricate constructs with tunable porosity, allowing precise control over drug release kinetics. The degree of porosity plays a critical role in modulating release profiles: higher porosity increases surface area and permeability, facilitating faster drug diffusion, while lower porosity enables sustained release by limiting diffusion pathways. The controllability of porosity is particularly advantageous in managing chronic conditions such as hypertension or diabetes, where controlled, long-term drug delivery is essential to maintain therapeutic concentrations and reduce dosing frequency. Furthermore, in wound healing applications, chitosan’s antibacterial properties and structural versatility support localized delivery of antibiotics or growth factors. By adjusting porosity in 3D-printed wound dressings, a gradual and sustained therapeutic release can be achieved, promoting tissue regeneration and infection control [87,88,151,152].

## 6. Conclusions and Perspectives

The integration of drug delivery systems and regenerative medicine has revolutionized healthcare by providing innovative solutions for treating a wide range of conditions, including neurodegenerative diseases, cardiovascular disorders, musculoskeletal injuries, and cancer. This review has explored key advancements in biomaterials, controlled drug release strategies, regenerative medicine, and tissue engineering, demonstrating their potential to enhance cell survival, targeted drug delivery, and functional tissue regeneration. By improving the precision and efficacy of therapeutic interventions, these technologies offer new hope for patients suffering from conditions that currently have limited treatment options.

Despite significant progress, several challenges remain, including scalability, cost-effectiveness, immune compatibility, and regulatory hurdles. Addressing these limitations is critical to ensuring the successful clinical translation of these therapies. Advances in 3D bioprinting, bioactive scaffolds, and patient-specific hydrogels have the potential to further refine regenerative strategies, particularly for osteochondral defects, chronic wounds, and organ failure, by enabling more precise tissue reconstruction and controlled drug delivery. The integration of expertise from materials science, bioengineering, pharmacology, and clinical medicine will be essential in overcoming existing barriers and accelerating the transition from laboratory research to clinical applications.

Furthermore, as cell-based and nanotechnology-driven therapies advance, regulatory frameworks must evolve to ensure safety, efficacy, and ethical compliance, especially in emerging areas such as gene editing, stem cell therapies, and biomaterial-based interventions. Personalized medicine is another promising avenue, where computational modeling and artificial intelligence can help optimize treatment strategies tailored to individual patients, improving outcomes for diseases such as cancer, diabetes, and spinal cord injuries.

Ensuring the sustainability and accessibility of these innovations is equally important. Making regenerative treatments affordable and scalable will be crucial for widespread adoption, particularly in resource-limited healthcare settings. By addressing these challenges and leveraging emerging opportunities, the integration of drug delivery and regenerative medicine will continue to drive transformative advancements in healthcare. These developments not only promise enhanced treatment efficacy and improved patient outcomes but also pave the way for a new era of personalized and regenerative medicine, ultimately redefining how diseases are treated and how damaged tissues are restored.

## Figures and Tables

**Table 2:** Materials for drug carriers, toxicity concerns, and degradation pathways.

Material	Toxicity Concerns	Metabolic Degradation Pathway	Reference
Liposomes	Mild immunogenicity, potential for allergic reactions	Enzymatic breakdown by phospholipases	[123]
Polyethylene Glycol (PEG)	Accumulation concerns, potential hypersensitivity reactions	Renal clearance (low MW) or liver metabolism (high MW)	[124]
Chitosan	Low toxicity; may trigger mild immune responses	Degraded by lysozyme and excreted via urine	[123]
Poly (lactic-co-glycolic acid) (PLGA)	Minimal toxicity, risk of acidic degradation byproducts	Hydrolyzed into lactic and glycolic acid, metabolized by liver	[125]
Dendrimers	Cytotoxicity, at high concentrations, affects cell membranes	Renal clearance for low MW, hepatic metabolism for high MW	[126]
Silica Nanoparticles	Potential oxidative stress, long-term accumulation risks	Partial degradation in lysosomes, excretion in urine	[127]
Gold Nanoparticles	Long-term retention in tissues, concerns over chronic toxicity	Minimal biodegradation, excreted slowly via the hepatobiliary route	[128]
Superparamagnetic Iron Oxide Nanoparticles (SPIONs)	Oxidative stress, potential liver accumulation	Degraded by lysosomes and cleared by macrophages	[126,129]
Hydrogels (e.g., alginate, collagen, hyaluronic acid)	Generally biocompatible, rare allergic reactions	Enzymatic degradation (e.g., collagenase, hyaluronidase)	[130]

## Data Availability

Not applicable.

## Abbreviations

API	active pharmaceutical ingredients
BMP	bone morphogenetic proteins
CDDP	cisplatin (a chemotherapy drug)
CH-GE	chitosan–genipin bioink
c-Myc	Cellular Myc
CXCR4	C-X-C Chemokine Receptor Type 4 (Chemokine receptor involved in signaling)
CPP	cell-penetrating peptide
DDSs	drug delivery systems
DOX	doxorubicin
DLP	digital light processing
ESCs	embryonic stem cells
ERT	Erlotinib
ECM	extracellular matrix
EHDA	electrohydrodynamic atomization
ES	electrospraying
EPR	enhanced permeation and retention
EVs	extracellular vesicles
FGFs	fibroblast growth factors
GI	gastrointestinal
GAGs	glycosaminoglycans
Hh	Hedgehog (regulates embryonic development, stem cells, and tissue patterning)
HMSNs	mesoporous silica nanoparticles
HA	hyaluronic acid
IGF-1	insulin-like growth factor
IVIVC	in vitro–in vivo correlation
iPSCs	induced pluripotent stem cells
Klf4	Kruppel-like factor 4
MSCs	mesenchymal stem cells
MHT	magnetic hyperthermia
MNPs	magnetic nanoparticles
MTE	multi-tip emitter
NPs	nanoparticles
NSCLC	non-small cell lung cancer
Notch	controls cell fate, stem cell maintenance, and differentiation
Oct4	Octamer-binding transcription factor 4
PLGA	poly (lactic-co-glycolic acid)
PCL	polycaprolactone
PEG	polyethylene glycol
PLA	poly(lactide)
PDA	polydopamine
PEGDA	poly (ethylene glycol) diacrylate
PCa	prostate cancer
SPIONs	Superparamagnetic Iron Oxide Nanoparticles
Sox2	[SRY (Sex-determining Region Y)-Box 2]
SMP	shape memory polymer
TMNPs	Trimagnetic Nanoparticles
Tg	glass transition temperature
VEGF	Vascular Endothelial Growth Factor
Wnt	Wingless/Int-1 (regulates cell growth, differentiation, stem cells, and tissue regeneration)
3D	three-dimensional
